# A Review of Global Patterns in Gut Microbiota Composition, Health and Disease: Locating South Africa in the Conversation

**DOI:** 10.3390/microorganisms13122831

**Published:** 2025-12-12

**Authors:** Nombulelo Mntambo, Thilona Arumugam, Ashiq Pramchand, Kamlen Pillay, Veron Ramsuran

**Affiliations:** 1 Department of Medical Microbiology, School of Laboratory Medicine and Medical Sciences, College of Health Sciences, University of KwaZulu-Natal, Durban 4041, South Africa; 220110399@stu.ukzn.ac.za (N.M.); cyborglona@gmail.com (T.A.); 216001715@stu.ukzn.ac.za (A.P.); 2 International Foundation for Integrative Medical Research, Cape Town 8001, South Africa; kamlenpillay@yahoo.co.uk; 3 Centre for the AIDS Programme of Research in South Africa (CAPRISA), University of KwaZulu-Natal, Durban 4013, South Africa

**Keywords:** gut microbiota, microbial diversity, dysbiosis, diet, health and disease, population variability, South African microbiome, urbanisation

## Abstract

The gut microbiota plays an essential role in human health through its contributions to immune regulation, metabolism, pathogen defence and disease susceptibility. Despite this significance, most gut microbiome research remains disproportionately focused on high-income countries, resulting in a limited and underrepresented view of global microbial diversity. This bias is evident in Africa, where populations, including those in South Africa, show unique combinations of genetic variation, dietary patterns and environmental exposures that are insufficiently captured in current datasets but offer opportunities to uncover novel insights into microbial evolution and its influences on health across diverse settings. In response to this gap, this review synthesises global patterns in gut microbiota composition and diversity while situating South African findings within this broader context. We examine evidence across microbial domains, including bacteria, fungi, viruses, archaea, protozoa and helminths, and highlight the impact of dietary transitions and environmental exposures on microbial community structure. Although still emerging, research on the gut microbiome of South African populations consistently reports contrasts between rural and urban populations, with rural groups enriched in fibre-fermenting and anti-inflammatory taxa, whereas urban communities often exhibit reduced diversity and features of dysbiosis linked to Westernisation. However, limited sample sizes, heterogeneous methodologies and absence of multi-omic approaches constrain robust interpretation. These lacunae in current knowledge emphasise the urgent need for large-scale, longitudinal studies that reflect South Africa’s demographic and geographic diversity. Strengthening this evidence will not only help identify microbial signatures linked to modifiable lifestyle factors but will also guide nutrition, prevention and screening programmes to improve health in African populations.

## 1. Introduction

The human gastrointestinal tract hosts a highly complex microbial ecosystem comprising bacteria, fungi, viruses, and archaea [1]. Estimated at nearly 100 trillion microorganisms, the gut contains the highest microbial density and diversity in the body, with approximately 500 to 1000 bacterial species, far exceeding the genetic repertoire of the human genome [2,3,4,5,6]. Although some microbial taxa are conserved across individuals, gut community structure varies substantially due to genetic background, diet and environmental exposures such as hygiene, sanitation, urbanisation and contact with diverse microbial reservoirs [7,8,9,10]. This variation extends beyond species-level classifications, with extensive strain-level and functional diversity shaping individual metabolic and immune responses [6,11,12,13,14].

Beyond its genetic complexity, the gut microbiota plays a critical role in physiological processes, including gut barrier integrity, brain development, nutrition, metabolism, digestion, vitamin synthesis, pathogen resistance and immune regulation, thereby maintaining overall homeostasis [15,16,17,18,19,20]. A stable and diverse gut microbiota has been reported to be associated with better health outcomes, whereas disruptions in this ecosystem, commonly referred to as dysbiosis, have been linked to metabolic diseases such as obesity, diabetes and cardiovascular disease [21,22,23,24,25,26,27,28]. However, there is no gold standard to define dysbiosis due to significant interindividual variation and the concept of a ‘healthy gut’ remains context-dependent [29,30,31]. Despite advances in microbiome research, the mechanisms driving gut microbial variation remain largely unknown, highlighting a critical knowledge gap [32].

Gut microbiome research remains disproportionately focused on Western populations, particularly European and North American cohorts, and predominantly targets industrialised populations with Westernised diets, limiting understanding of microbial diversity in non-Western cultural and dietary contexts [33,34,35]. Africa, accounting for nearly 19 percent of the global population, is significantly underrepresented in human microbiome studies, especially in regions with high population growth [36]. Existing gut microbiome research in African communities has mainly focused on childhood undernutrition, infectious diseases or traditional subsistence lifestyles [37,38,39,40]. These studies consistently show an inverse relationship between the relative abundances of *Bacteroides* and *Prevotella*, with *Bacteroides* being more dominant in Western diets rich in animal fat and protein and *Prevotella* more prevalent in plant-based diets in non-Western populations [41,42,43]. While data on healthy African individuals remain sparse, these studies provide valuable insights into microbiome composition in traditional communities, although the health implications of these variations are not yet fully understood [44].

Non-communicable diseases such as cancers, diabetes, obesity and cardiovascular conditions are major public health concerns globally [45,46]. In South Africa, these challenges are further compounded by a quadruple burden of disease, including HIV/AIDS and tuberculosis, elevated maternal and child mortality, widespread violence and injury-related health impacts, and the rising prevalence of non-communicable diseases [47,48,49,50]. These overlapping health complications affect both rural and urban populations, contributing to a multifactorial public health paradigm [51,52].

Moreover, these challenges are exacerbated by an ongoing epidemiological transition, with rising lifestyle-related chronic diseases coexisting alongside persistent infectious disease burdens [53,54]. Urbanisation, dietary shifts toward processed foods, and socioeconomic disparities further drive these trends [55,56,57,58]. Despite these pressing health concerns, the role of the gut microbiota in disease prevention and management among African populations remains largely underexplored [59]. This knowledge gap limits understanding of how microbial imbalances may influence Africa’s dual burden of communicable and non-communicable diseases, given the growing evidence supporting the gut microbiota’s role in metabolism, immunity, and inflammation.

To fully leverage gut microbiome research for public health in African settings, it is imperative to characterise the composition and functional dynamics of the human gut microbiota across diverse populations [37,60], including major ethnolinguistic groups in South Africa such as Nguni (Zulu, Xhosa, Swazi, and Ndebele), Sotho-Tswana (Southern Sotho, Northern Sotho, and Tswana), Venda, Tsonga, as well as South Africans of Indian, Coloured, and European descent [61]. These populations exhibit distinct genetic backgrounds, dietary habits, and environmental exposures, which shape microbiome composition and influence disease susceptibility [39,62].

The scarcity of region-specific data presents a significant barrier to developing targeted medical interventions that account for varied dietary practices, socio-environmental contexts, and the country’s unique epidemiological landscape [63,64]. Given the limited representation of African populations in microbiome research, this literature review aims to examine gut microbiota composition, explore its associations with health and disease, and situate South African findings within the broader global context.

## 2. From Colonisation to Composition: Factors Shaping Gut Microbial Communities

The human gastrointestinal tract is home to a vast and diverse ecosystem shaped by many chemical, physical and environmental factors as it extends from the mouth cavity to the stomach, continues through the intestines and ultimately reaches the anus [65,66]. This ecosystem consists of a complex and dynamic community of microbes, including bacteria, viruses, fungi, and archaea, that begin colonising the digestive system shortly after birth [67,68]. The microbiome’s composition varies as it traverses the gastrointestinal tract, with dominant microbial populations characterised by distinct functional roles at different sites. Variations are largely influenced by factors such as pH, oxygen levels, transit time and nutrient availability [69]. While microbial presence is found throughout the gastrointestinal tract, studies have shown that colonisation is most concentrated in the intestines, particularly the colon, where the gut microbiota plays a crucial role in digestion, immunity and overall health [17,70,71].

The long-standing relationship between humans and their gut microbiota has led to the coevolution of microorganisms, resulting in adaptive traits that benefit both the microbes and their host [72,73]. Milani et al. (2017) [10] and Loo et al. (2019) [74] describe each individual as a distinct microenvironment that harbours a unique microbial community, influenced by specific ecological principles that govern its structure and diversity [10,74]. Building on this understanding, Kijner et al. (2022) [75] provided evidence that these microbial populations are established during infancy and continue to develop into adulthood [75]. This early colonisation is shaped by many factors, including the mode of delivery, feeding practices such as breastfeeding or formula feeding, weaning, and the maternal microbiota [76,77,78]. The interactions between the early-life microbiome and the host’s developmental pathways may profoundly shape long-term health outcomes [79]. Even in the presence of shared early-life influences, the composition of the gut microbiota is highly individualised, with distinct differences observable between mothers and their infants soon after birth [80].

What begins as a shared developmental process soon gives rise to uniquely structured microbial communities that persist throughout one’s lifetime. Factors such as diet, environmental exposures and pharmaceutical interventions, particularly antibiotic use, have been shown to have significant, and at times disruptive effects on microbial composition, contributing to pronounced interindividual variation [81]. However, the emphasis on variability should not obscure emerging evidence of microbial similarities among individuals with shared characteristics. Studies have reported compositional overlaps linked to age [82,83], dietary profiles [28,84,85], ethnicity [86,87], geographic location [88,89,90] and even genetic background [8,91]. Taken together, these findings contest the notion that microbial assembly is random and instead point to a multifactorial framework in which both internal (host-related) and external (environmental or cultural) factors co-produce distinct microbial patterns.

While earlier studies posited a stronger role for host genetics in shaping gut microbiota composition, Rothschild et al. (2018) [6] challenged this view by demonstrating that environmental factors had a stronger influence. People who were not genetically related but shared a household had a more similar gut microbiota profile than relatives living separately, underscoring the dominant role of shared environmental factors like diet and lifestyle [6]. Echoing these findings, Benson et al. (2010) [92] acknowledged a genetic role in gut microbiota composition but highlighted the stronger influence of environmental factors [92]. However, more recently, Zhernakova et al. (2024) [93] demonstrated that, although broad patterns of microbiota composition are predominantly shaped by environmental factors, certain genetic loci are nonetheless associated with specific microbial taxa [93]. Rather than supporting a strict divide between nature and nurture, these insights reveal a more entangled relationship, one in which genetic and environmental factors are intertwined.

**Figure 1:**
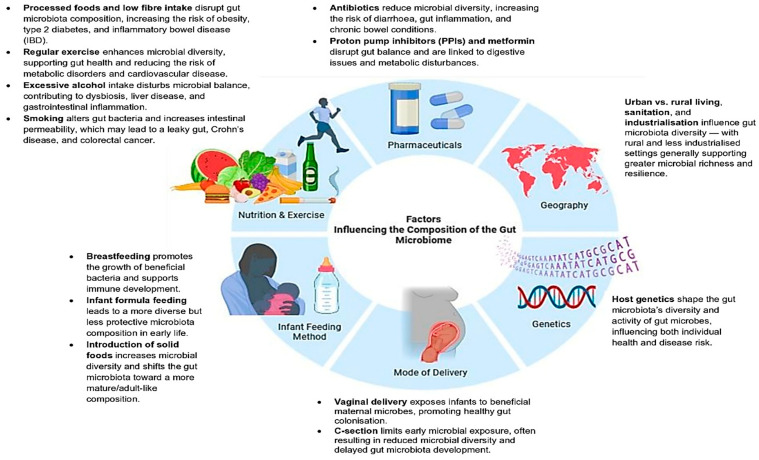
Factors influencing the composition of the gut microbiome across the human life course and their implications for health and disease.

Beyond the foundational roles of genetics and lifestyle, increasing attention has turned to how specific exposures across the life course shape and sometimes disrupt gut microbial ecosystems [94]. As shown in Figure 1, factors such as mode of delivery, infant feeding practices, diet and exercise, geographic environment, sanitation, medication use and lifestyle habits like smoking act as key modulators of gut microbiota composition, some exerting short-term effects, while others lead to lasting microbial changes with implications for health and disease [95,96]. These disturbances are highly context-dependent and may compound over time, contributing to variability in gut microbial profiles across individuals and populations [97]. Importantly, much of the available literature originates from high-income countries, frequently overlooking region-specific exposures such as dietary patterns, living conditions and pathogen prevalence that may uniquely influence microbial dynamics in African populations.

Expanding on the complexities of microbiota–host interactions, recent studies have also explored how distinct microbial alterations may underpin susceptibility to or progression of various diseases. For instance, diminished microbial diversity is frequently observed in metabolic syndromes and inflammatory bowel diseases, while certain cancers and autoimmune disorders are characterised by the expansion of opportunistic pathogens or the depletion of protective taxa [98,99]. However, questions of causality remain unclear: whether these microbial shifts precede disease onset or arise as a consequence of pathological changes is still under investigation [100,101]. This uncertainty complicates efforts to develop precise microbiome-targeted interventions and reinforces the importance of understanding how microbial composition is shaped across the lifespan through a continuous interplay of colonisation events, host biology, environmental exposures and external disruptions.

## 3. Microbial Diversity Beyond Bacteria: The Neglected Non-Bacterial Constituents of the Gut Microbiome

The current understanding of the gut microbiota is heavily skewed toward bacterial communities, which have been the primary focus of most microbiome research [102]. In contrast, other microbial domains such as viruses, archaea, fungi, protozoa and helminths remain comparatively underexamined due to longstanding technical and conceptual challenges. For example, standard DNA extraction protocols optimised for bacteria often prove inadequate for fungi, whose thick chitin-rich cell walls impede efficient lysis and sequencing [103,104]. Despite these limitations, the gastrointestinal tract is now recognised as the most thoroughly studied fungal environment in humans, though fungal diversity still pales in comparison to that of bacteria. Archaea are even less understood, owing to a historical lack of prioritisation, limited reference genomes and methodological barriers that complicate detection and characterisation [105]. Similarly, protozoa (unicellular eukaryotes such as *Blastocystis* and *Entamoeba*) and multicellular helminths have long been viewed through a pathogenic lens, often overlooked in microbiome studies despite growing evidence of their potential commensal or modulatory roles. On the other hand, the gut virome (particularly bacteriophages) also remains under-characterised despite its abundance and potential role in regulating microbial ecology and host health. Efforts to map this domain are hampered by difficulties in viral genome annotation and the high variability of viral communities [106,107]. Addressing these gaps is essential for a more integrative and accurate picture of human gut ecology. In this section, we examine the roles and diversity of non-bacterial components of the gut microbiome, including fungi, viruses, archaea, protozoa, and helminths.

### 3.1. Fungi (The Mycobiome): Understudied Immune Modulators

The gastrointestinal tract hosts a diverse yet comparatively understudied fungal community, collectively referred to as the gut mycobiome [108]. Despite technical barriers such as the chitinous structure of fungal cell walls that complicate DNA extraction [109], this environment is currently regarded as the most examined fungal niche in the human body [110]. Nevertheless, the diversity, stability and functional relevance of the gut mycobiome remain far less understood than that of bacteria [103,104]. Fungal diversity is also substantially lower than bacterial diversity, with dominant phyla including Ascomycota, Basidiomycota, and Zygomycota, and commonly reported genera such as *Candida* (notably *Candida albicans*), *Saccharomyces*, *Penicillium*, *Aspergillus*, *Cryptococcus*, *Malassezia*, *Cladosporium*, *Galactomyces*, *Debaryomyces*, and *Trichosporon* [111,112,113].

Among these, *C. albicans* is of particular interest due to its opportunistic behaviour and association with dysbiosis. Elevated levels of *Candida* have been reported in individuals with irritable bowel syndrome (IBS) and Crohn’s disease, where they may impair intestinal barrier function leading to a “leaky gut”, provoke inflammation and contribute to visceral hypersensitivity [114,115,116]. Apart from their association with disease, fungi also play a regulatory role through their interactions with bacterial communities and the immune system. These cross-kingdom interactions affect the production of short-chain fatty acids (SCFAs) which are key molecules for gut homeostasis and host metabolism [117].

Most insights into the gut mycobiome have emerged from Western cohorts, where studies frequently report high prevalence of *Candida*, *Saccharomyces* and *Malassezia*, often linked to Westernised diets and higher rates of obesity and inflammatory bowel disease. African studies, although limited, suggest distinct patterns. In Cape Town, Nel Van Zyl et al. (2022) [118] analysed the gut mycobiota of 115 young children and found *Candida* and *Saccharomyces* to be dominant, with *Candida* more abundant in children under two. Fungal diversity was lower than bacterial diversity and associated with vitamin A supplementation and microbial cross-domain interactions. Although only one participant was living with HIV, the study did not report distinct fungal profiles or clinical implications for this individual [118]. Nonetheless, the findings highlight the potential for unique fungal signatures influenced by nutritional status and environmental exposures in African populations.

While the Cape Town study focused on children, complementary work by Kabwe et al. (2020) [119] examined the gut mycobiota of 100 healthy adults from rural villages in Limpopo and the city of Pretoria, Gauteng. In this cohort, *Pichia* emerged as the most common genus across participants, representing the highest relative abundance among the fungi identified. Its prevalence was higher in non-smokers compared to smokers, suggesting a possible sensitivity to tobacco-related exposures. Conversely, *Candida tropicalis* was more abundant in smokers, while *Saccharomyces cerevisiae* was more frequently found in non-smokers [119]. These patterns align with previously reported antagonistic interactions among fungal species, though further research is needed to validate these associations in larger populations. The study did not directly examine dietary influences, but the authors referenced earlier work showing links between dietary patterns such as vegetarian versus conventional diets and shifts in fungal composition [120,121]. Overall, these findings suggest that *Pichia* may play an important role in the South African gut mycobiome and emphasise the importance of including detailed lifestyle and health data in future studies. Collectively, the limited data available imply that fungal communities in South African groups could be shaped by a complex interaction of age, environmental exposures and behaviours like smoking, underscoring the need for large-scale, population-based studies to better understand the mycobiome’s influence on health and disease.

### 3.2. Viruses (The Virome): An Underexplored Driver of Microbial and Immune Dynamics

The gut virome, comprising bacteriophages and eukaryotic viruses, plays a crucial but often overlooked role in regulating microbial ecology and modulating host immunity [122]. Disruptions to this viral ecosystem are increasingly recognised for their broader impact on gut homeostasis and systemic inflammation, being associated not only with HIV but also with conditions such as inflammatory bowel disease (IBD) [123]. Within this complex viral community, bacteriophages have been reported to influence bacterial population dynamics and facilitate gene transfer, while eukaryotic viruses, such as anelloviruses, have been linked to immune modulation [124].

Recent work by Hetta et al. (2025) [125] reports that the gut virome should not be considered a “minor” component of the microbiome: although viruses represent a small fraction of total microbial biomass, they often outnumber bacterial cells and can exert a substantial influence on microbial ecology, immune interactions, and disease processes [125]. Moreover, it has been reported that the gut virome exhibits alterations in viral community composition IBD, including expansions or shifts in specific bacteriophage populations and changes in eukaryotic viruses [126]. These changes may influence gut barrier integrity, immune signalling, and interactions with the bacterial microbiome, although causal relationships remain to be established [127].

Building on this broader understanding of virome–host interactions, studies have shown that in people living with HIV, levels of anelloviruses tend to be higher in individuals with low CD4^+^ counts and decrease with immune recovery, indicating their potential as biomarkers of immunological status and treatment response [128]. Similarly, research in a Ugandan cohort has demonstrated that advanced HIV is associated with increased enteric adenoviruses and reduced bacterial diversity in the gut microbiome, with immunosuppression rather than antiretroviral therapy (ART) being the primary factor driving these virome alterations [129]. Despite differences in geography and viral taxa, both studies demonstrate that HIV-related immunosuppression profoundly disrupts the gut virome and bacterial microbiome, indicating immune status as a central determinant of gut microbial and viral community structure across populations.

In South Africa, shotgun metagenomic profiling of gut microbiomes in rural (Bushbuckridge) and urban (Soweto) adult women revealed notable patterns in both bacterial and viral communities [130]. While this study primarily focused on bacterial composition and the identification of novel taxa, the sequencing approach also captured viral DNA, including crAssphage and crAss-like phages. CrAssphages are bacteriophages that infect *Bacteroides* species and play a key role in shaping bacterial populations and maintaining gut ecosystem balance while CrAss-like phages are a genetically related, more diverse group of bacteriophages that share similar ecological roles in the gut [131]. In these cohorts, crAssphages and crAss-like phages were more abundant in urban Soweto samples, suggesting geography-associated viral shifts. These phages are generally more abundant in Westernised gut microbiomes, influenced by diets high in fat and protein [130]. Although associations between crAssphage abundance and specific dietary categories are generally weak, broader trends indicate that their prevalence may have increased alongside urbanisation and Western dietary transitions [132]. Moreover, these phages are prevalent in many global cohorts but appear relatively underrepresented in African datasets, likely reflecting the limited availability of shotgun metagenomic data rather than a true absence [130,132].

By contrast, a virome-focused study in urban (Khayelitsha, Cape Town) and rural (Zithulele Hospital, Eastern Cape) Xhosa adults combined targeted viral metagenomics with metabolomic profiling, revealing over 900 viral contigs, mostly double-stranded DNA viruses, of which approximately 82% could not be classified, highlighting the extensive uncharted viral diversity in African populations [34]. Although the differences were not statistically significant, rural participants tended to have higher levels of Podoviridae and crAssphages, while urban participants showed modest increases in Siphoviridae, Microviridae, and temperate phages. This pattern differs from trends observed in other studies, which indicate greater crAssphage abundance in urban or Western cohorts [130,132].

Moreover, this inconsistency may reflect differences in local diets, environmental exposures or methodological approaches between studies. It also emphasises the need for standardised virome investigations across populations to accurately characterise viral diversity and its potential health implications. Viral communities clustered distinctly by geography were correlated with host bacterial groups, such as Oscillospiraceae in rural areas and *Faecalibacterium* spp. like *Faecalibacterium prausnitzii* in urban individuals, as well as with diet-associated metabolites, including bile acids and vitamins. Rural diets were richer in fibre, associated with elevated levels of butyric acid, aconitate, and α-ketoglutarate [130]. Interestingly, genera of the Oscillospiraceae family *Faecalibacterium*, *Oscillibacter* and *Ruminococcus*, which have previously been reported in urban Western populations [133], were also observed in a rural South African cohort [130]. This raises questions about its ecological role in high-fibre, rural diets and whether its presence in Africa reflects a conserved functional role in gut metabolism or potential early markers of metabolic shifts. These microbial patterns aligned with early metabolic indicators of colorectal cancer (CRC) risk, including increased secondary bile acids and reduced microbial diversity [133].

Contrarily, urban diets which are typically higher in fat, cholesterol and animal protein, have been shown to promote bile-adapted bacteria such as *Lachnoclostridium* [134]. These urban dietary and microbial patterns aligned with early metabolic markers of CRC risk, including increased secondary bile acids and reduced microbial diversity, reinforcing concerns about the health impacts of rapid dietary Westernisation [135,136].

While microbiome research in South African adults has predominantly focused on bacterial communities using 16S rRNA or shotgun metagenomic sequencing, the work by Ramaboli et al. (2024) [34] represents the most comprehensive attempt to date to characterise the gut DNA virome in an adult South African cohort. By comparison, existing virome studies in the region have focused mainly on paediatric populations (e.g., [137]) or on animal reservoirs such as poultry and pigs [138,139], leaving the adult human virome largely uncharted.

### 3.3. Archaea: Methane Producers and Hydrogen Scavengers

Methane-producing archaea, notably *Methanobrevibacter smithii* and *Methanosphaera stadtmanae*, constitute a unique and metabolically active fraction of the gut microbiota where their involvement in hydrogen turnover and methanogenesis enhances fermentation dynamics and overall ecosystem balance [140]. However, despite these beneficial functions, elevated levels of these archaea, particularly *M. stadtmanae*, are associated with inflammatory conditions such as inflammatory bowel disease [141]. Through the fermentation of hydrogen and carbon dioxide, these microbes generate methane: a process that influences gut physiology and microbial interactions [142]. Notably, previous work has shown that although archaeal diversity in the human distal gut is relatively low, these communities tend to be highly individual-specific and remain temporally stable. This suggests that archaea, despite their limited diversity, constitute a persistent and potentially foundational component of the gut ecosystem [122].

The functional repertoire of gut archaea continues to expand, challenging earlier assumptions of their limited diversity and ecological influence. Gaci et al. (2014) [143] drew attention to the emerging role of the archaeal order *Methanomassiliicoccales*, a lineage phylogenetically and metabolically distinct from classical hydrogenotrophic methanogens. Unlike *M. smithii*, which relies on hydrogen and carbon dioxide for methane production, *Methanomassiliicoccales* utilise methylated compounds such as trimethylamine (TMA), a metabolite derived from dietary precursors like choline and carnitine [143]. This alternative methanogenic pathway has sparked interest for its clinical relevance, as it may reduce systemic levels of trimethylamine-N-oxide (TMAO), a pro-atherogenic compound implicated in cardiovascular disease [144]. The presence of these methylotrophic archaea not only broadens our understanding of archaeal metabolic plasticity but also introduces the concept of “archaebiotics”, archaea-based interventions aimed at mitigating host metabolic dysfunction.

However, interpretations of archaeal prevalence and function must be approached with caution. Discrepancies in archaeal detection across microbiome studies are often artefacts of differing DNA extraction protocols, primer sets, or sequencing platforms [140]. Technical limitations aside, ecological factors such as diet-driven niche competition also shape archaeal dynamics. For example, *Methanobrevibacter* and *Nitrososphaera*, two commonly reported gut archaea, appear to be mutually exclusive in certain host populations, potentially reflecting competition for ecological niches or divergent dietary associations [145,146]. Evidence indicates that *Methanobrevibacter* abundance correlates positively with high-carbohydrate diets and inversely with intake of amino acids, proteins, and fatty acids, illustrating the dietary sensitivity of archaeal colonisation [147]. These findings not only illustrate the dietary sensitivity of archaeal colonisation but also point to an underexplored axis of microbe–microbe and microbe–host interactions that may be central to gut ecosystem stability.

The potential role of archaea in gut pathology is further illustrated by their dynamic shifts in inflammatory conditions such as Crohn’s disease (CD) [148]. A notable reduction in *M. smithii* alongside a striking three-fold increase in *M. stadtmanae* has been reported in individuals with CD, with levels returning to baseline during remission, highlighting their sensitivity to inflammatory states [149]. This fluctuation suggests that archaea may be more than passive residents, potentially contributing to disease activity. One proposed mechanism linking archaeal shifts to chronic inflammation is the “syntrophic imbalance hypothesis” [150]. This theory suggests that SCFAs, particularly butyrate, are essential for maintaining the structural and functional integrity of microbial biofilms, including those involving archaea. In dysbiotic states, excessive SCFA depletion may destabilise these biofilms, leading to unchecked archaeal proliferation and enabling bacteria to transition from commensal to invasive phenotypes. Such translocation into epithelial tissue is thought to exacerbate intestinal inflammation, establishing a feedback loop that perpetuates mucosal injury [151].

Despite the growing recognition of archaea as metabolically active and immunologically relevant members of the gut microbiome, data on their presence and functional roles within African populations remain virtually absent. While studies from Europe, North America and parts of Asia have characterised key archaeal taxa like *M. smithii* and *M. stadtmanae*, comparable efforts in African contexts are lacking. To date, no published South African human gut microbiome study has intentionally profiled archaea, either through targeted amplicon sequencing or metagenomic reconstruction. This gap is notable given the continent’s high burden of diseases such as HIV, IBD, and malnutrition; conditions in which archaeal populations have been shown in other settings to influence key aspects of gut physiology. For example, methanogenic archaea such as *M. smithii* have been associated with substantially elevated methane emission and shifts in gut metabolites, including increased formate and acetate, highlighting archaeal contributions to metabolic regulation within the gut [152].

In addition, archaeal communities show age-associated variation; methanogen abundance and composition change across the lifespan, indicating that archaeal populations are dynamic and responsive to physiological states [153]. While no studies to date have characterised the gut archaeome in the context of HIV infection or malnutrition, work in IBD has demonstrated that the absence or reduction in *Methanobrevibacter* within ileocolonic biofilms corresponds with disrupted gastrointestinal homeostasis, increased facultative anaerobes, reduced microbial diversity, and altered bile acid metabolism, suggesting that archaea may play a stabilising role within the mucosal ecosystem [154]. Although archaea are unlikely to serve as standalone diagnostic markers, profiling them can reveal metabolic, ecological, and immunological mechanisms that are not captured by bacterial analyses alone, thereby offering complementary insights into microbial contributions to disease susceptibility and progression [142]. Incorporating archaeal-targeted primers or metagenomic approaches optimised to recover archaeal genomes would therefore provide a more complete characterisation of the gut microbiome and may help clarify microbial contributions to health and disease in African populations.

### 3.4. Protozoa and Helminths: From Pathogens to Partners in Immune Regulation?

Protozoa and helminths are frequently excluded from standard gut microbiome analyses, likely due to their historical framing as parasitic threats to human health [155]. However, findings from at least one African study suggest that their ecological roles may extend beyond pathogenicity, potentially influencing host immunity and gut microbial interactions [156].

Recent studies have challenged long-standing assumptions that *Blastocystis* is merely a pathogenic protist. In a rural Mexican cohort, its presence was associated with increased bacterial alpha diversity, reduced *Prevotella copri*, and elevated levels of Clostridia such as *Ruminococcus bromii*, alongside increases in several fungal taxa, unique metabolic profiles, and reduced intestinal inflammation [157]. These findings suggest a potentially symbiotic role, at least in certain environmental or dietary contexts.

A French cohort similarly reported higher bacterial diversity and enrichment of Ruminococcaceae in individuals colonised with *Clostridioides difficile*, although this was accompanied by contrasting increases in Prevotellaceae [158]. This regional divergence was echoed in Danish data, where *Blastocystis* colonisation correlated with greater *Prevotella* and reduced *Bacteroides* abundance [159]. A global meta-analysis found *Prevotella copri* was commonly associated with colonised individuals, whereas *Bacteroides* species and *Ruminococcus gnavus* were enriched in those without *Blastocystis* [160]. These contrasting results across populations suggest that *Blastocystis*’ ecological role is not fixed but shaped by socio-geographic factors, host diet and microbial context. Subtype-specific differences may also underlie the observed variability.

Helminths also present a more multifaceted case than previously assumed. In Uganda, investigations into *Schistosoma mansoni* infections in rural fishing communities found distinctive microbial alterations among infected individuals. Notably, these shifts were accompanied by reduced hepatitis B vaccine responsiveness, suggesting that helminth-induced immune conditioning may lower the effectiveness of immunisation efforts in endemic areas. These microbial and immunological effects occurred in the absence of overt pathology, lending weight to the idea that helminths may at times engage in functionally symbiotic rather than strictly pathogenic interactions, subverting the common categorisation of protozoa as purely harmful and instead pointing to localised microbial ecosystems where these organisms may coexist beneficially with the host [156].

South African studies add further texture to this evolving narrative. In Mthatha, Eastern Cape, a high prevalence of helminth infections was reported among individuals living with HIV, with worm burdens significantly associated with lower CD4^+^ T-cell counts [161]. Although microbiome profiling was not conducted, the immunological implications of this coinfection are clear and they lend localised support to broader conclusions drawn from systematic reviews of helminth–HIV/TB coinfection literature across Sub-Saharan Africa [162]. These reviews found substantial evidence linking helminths to increased immune activation, impaired TB-specific Th1 responses, elevated viral loads, diminished CD4^+^ counts, and heightened risks of anti-retroviral treatment (ART) failure and mother-to-child transmission. While some studies reported no adverse effects, the overall weight of the evidence suggested that helminth coinfection may exacerbate HIV-related immune dysfunction. Although narrower in scope, the findings from this South African cohort empirically reinforce the conclusion that helminth burden should be considered in HIV clinical management, particularly in endemic, socioeconomically vulnerable settings like the Eastern Cape [161]. Together, these studies highlight the importance of integrating parasitological surveillance and deworming strategies within HIV care frameworks to mitigate immunological compromise [162].

Further work in peri-urban KwaZulu-Natal explored the intersection of HIV and helminth infections among adults, revealing how structural and socioeconomic vulnerabilities shape patterns of disease [163]. Coinfections were particularly common among individuals from low-income households, those lacking proper sanitation infrastructure, and older adults. Interestingly, *Ascaris lumbricoides* emerged as the dominant helminth species in this population [163]. These findings suggest that the persistence of dual infections in these communities reflects not only biological susceptibility but also entrenched inequalities in housing, sanitation, and healthcare access, reinforcing the need to address social determinants of health when designing integrated intervention strategies for populations burdened by overlapping infections.

What sets these African studies apart is their consistent challenge to the one-dimensional view of helminths and by extension, other gut eukaryotes such as protozoa are inherently pathogenic [164]. Instead, they suggest a spectrum of interactions ranging from disruptive to potentially protective, depending on host, microbial and environmental context. This contrasts with dominant Western biomedical models, which often overlook the long evolutionary history of host–parasite coexistence in non-industrialised settings [165,166]. Moreover, the lack of sufficient gut microbiota data in many South African studies highlights a significant research gap because without integrated parasitological, microbial and immunological analyses, the full ecological roles of these microorganisms in the gut remain elusive.

### 3.5. Interactions Between Microbial Components

In essence, the gut microbiota forms a dynamic and interconnected network of microorganisms that engage in complex interactions to influence host physiology and immune function [3]. These microbes communicate through various mechanisms such as chemical signalling, metabolic exchanges, and direct physical contact [85]. For example, bacteria use quorum sensing (QS), a cell–cell communication mechanism that relies on the secretion and detection of signalling molecules called autoinducers, to coordinate collective behaviours such as biofilm formation and virulence expression [167]. Viruses, particularly bacteriophages that infect bacteria, play a key role in modulating bacterial populations not just through lysis but also by facilitating horizontal gene transfer, thereby increasing microbial diversity and adaptability [168,169]. Simultaneously, fungi within the gut mycobiome produce bioactive compounds and metabolites that either inhibit or promote bacterial growth, shaping microbial balance and impacting host health [170].

Archaea, though less abundant, participate in essential metabolic activities such as hydrogen consumption and methane production, which influence bacterial fermentation pathways and host energy metabolism [143]. These inter-kingdom interactions are further affected by environmental factors such as pH, nutrient availability and oxygen levels; all of which add to the complexity and stability of the gut ecosystem [171]. Helminths and protozoa, long framed as pathogens, are increasingly recognised as modulators of gut ecology, capable of shaping bacterial, fungal and immune landscapes in context-dependent ways [156]. Studies across diverse populations reveal associations between these eukaryotes and increased microbial diversity, altered metabolite profiles and shifts in dominant bacterial taxa such as *Clostridia* and *Prevotella*, suggesting potential roles in immune regulation and microbial stability [157]. Understanding these detailed microbial dialogues is vital, as disturbances, whether from infections, antibiotics or dietary changes can disrupt community structure and function, making the host susceptible to various diseases.

These complex inter-kingdom interactions between bacteria, archaea, viruses, fungi, protozoa and helminths reflect the intricate and co-dependent nature of the gut ecosystem [172]. Yet, much of this evidence stems from research in high-income countries, leaving significant gaps in understanding how these dynamics unfold in African populations. Given South Africa’s unique genetic diversity, dietary patterns, environmental exposures and disease burdens, investigating the full spectrum of gut microbial life in this region is essential. Studies that isolate one microbial domain risk presenting an incomplete or narrow image of microbial ecology and its relevance to health and disease. Table 1 provides a comparative overview of microbial groups by summarising their typical abundance patterns, dominant genera, core functional roles, and associated disease outcomes. Importantly, the table integrates genera commonly reported across both Western and African populations, offering a consolidated perspective that reflects globally observed patterns while highlighting context-dependent variation. This combined framework reinforces the need for a more integrated and holistic approach to microbiome research.

## 4. From Harmony to Disruption: The Immunological Cost of Dysbiosis

The gut microbiota plays a pivotal role in shaping immune responses and influencing susceptibility to metabolic and inflammatory diseases. Commensal microbes reinforce intestinal barrier integrity by stimulating mucin and tight junction protein production and by promoting the secretion of antimicrobial peptides (AMPs) by Paneth cells, thereby limiting pathogen translocation [173]. Microbial metabolites, particularly short-chain fatty acids such as butyrate, contribute to immune regulation by promoting regulatory T cell differentiation and dampening pro-inflammatory Th17 responses [174]. The microbiota also supports colonisation resistance and modulates host immunity through inflammasome and cytokine signalling [175,176].

Early-life microbial colonization, disrupted by caesarean delivery, antibiotics, or formula feeding; has lasting consequences for immune development and disease susceptibility [177]. When this equilibrium is disrupted, a state termed dysbiosis, the consequences span a wide range of metabolic, inflammatory, and gastrointestinal disorders [178]. Dysbiosis can induce chronic low-grade inflammation, impair gut barrier function, and alter metabolic signalling pathways, contributing to conditions such as obesity, insulin resistance, CVD, type 2 diabetes, IBD and IBS [179]. Key taxa like *Faecalibacterium* and *Akkermansia* are associated with anti-inflammatory states and improved metabolic outcomes, whereas an overabundance of *Proteobacteria* may signal inflammation and systemic immune activation [180].

In the case of IBD, emerging evidence highlights that dysbiosis involves not only compositional changes but also functional and metabolic alterations [181]. Reductions in short-chain fatty acids, particularly butyrate, are consistently observed in IBD and correlate with impaired epithelial barrier integrity, decreased regulatory T-cell induction, and increased pro-inflammatory signalling [182]. Dysbiotic shifts may also favour expansion of facultative pro-inflammatory taxa, such as Enterobacteriaceae, while depleting obligate anaerobes that perform fibre fermentation and produce immunomodulatory metabolites. This integrative perspective suggests that the pathogenesis of IBD cannot be attributed solely to taxonomic shifts but must consider the combined impact of microbial composition, metabolic output, and host immune interactions [181].

Microbial metabolites also serve as mechanistic links to disease. For example, trimethylamine-N-oxide (TMAO), a by-product of microbial metabolism of choline and carnitine, has been implicated in endothelial dysfunction and atherosclerosis [183]. Additionally, in IBS, reduced fungal diversity and overgrowth of *Candida* species may exacerbate symptoms [184,185]. Despite efforts to define dysbiosis, findings remain heterogeneous and sometimes contradictory, particularly regarding biomarkers like the Firmicutes-to-Bacteroidetes (F/B) ratio. Some studies report increased F/B ratios in obesity, while others show no clear trend [186,187]. These inconsistencies highlight the need for multifactorial and context-specific assessments that integrate microbial diversity, functional potential, and host interactions especially in low- and middle-income countries.

Therapeutic strategies aimed at restoring microbial balance such as faecal microbiota transplantation (FMT), probiotics and prebiotics, are being actively explored. FMT has shown particular success in treating recurrent *Clostridioides difficile* infections by restoring microbial diversity [116]. While taxa such as *Faecalibacterium* are broadly linked to health in Western cohorts, their role and prevalence in African populations require further study. These dynamics are especially urgent in South Africa, where rising rates of metabolic and inflammatory diseases [48] coexist with infectious conditions such as HIV and TB. Widespread antibiotic exposure [188], increasing dietary Westernisation [189] and persistent food insecurity [190] intersect within a socioeconomically vulnerable population. Nevertheless, efficacy and mechanistic understanding remain limited outside controlled contexts.

Overall, while the literature provides compelling models for microbial influence on immune and metabolic health, significant gaps remain. Many findings are derived from Western-centric cohorts, and heterogeneity in study design, environmental exposures, and analytic approaches complicates generalisability. A critical, context-sensitive evaluation that integrates microbial composition, function, and host interactions is therefore essential to advance understanding of gut microbiome impacts on health and diseases. To contextualise these disruptions, we describe below the evolving concept of dysbiosis: its definitions, variability, and relevance to underrepresented populations exposed to shifting health and environmental conditions.

### Defining Dysbiosis and Its Variability

Under normal conditions, the gut microbiota is relatively stable and resilient, fostering a symbiotic relationship with the host [191]. Dysbiosis, often defined as an imbalance, disruption, or loss of microbial diversity, is increasingly recognised as a marker of poor health [192,193]. However, establishing a universal definition remains challenging due to significant interindividual variability influenced by factors such as genetics, geography, diet, lifestyle, age, medication use and environmental exposures [194,195]. Although widely used in the literature, the term ‘dysbiosis’ is a broad descriptor of microbial alterations that deviate from a presumed “normal” or reference microbiota [26,196,197]. Yet not all such deviations are inherently harmful. Some microbial shifts may be neutral or even beneficial, depending on the host’s context. This complexity is evident in cases where microbial shifts yield therapeutic benefits. For instance, metformin-induced alterations in gut microbiota have been associated with improved glycaemic control and enhanced therapeutic efficacy [198,199,200]. The concept of “good dysbiosis” accentuates the complexity of microbiome–host interactions and cautions against overly simplistic classifications.

A higher microbial diversity is generally associated with better health, provided the ecosystem remains balanced [201,202]. Several indices beyond traditional alpha and beta diversity metrics have been developed to more precisely characterise dysbiosis, offering insights into disease prediction and treatment responses. These include Shannon’s Index (diversity), Simpson’s Index (dominance), and Pielou’s Evenness (distribution) as well as more advanced metrics like UniFrac distances and the Functional Gene Index which assess phylogenetic relatedness and functional shifts in microbial communities [26,201,203]. Furthermore, urbanisation, dietary transitions, antibiotic exposure and changing hygiene practices have contributed to global shifts in microbial composition, especially in low- and middle-income countries, altering microbial resilience and potentially predisposing individuals to non-communicable diseases [10,188,189,190]. As such, the definition and implications of dysbiosis may differ depending on regional and population-level baselines, highlighting the need for context-specific reference frameworks and the inclusion of underrepresented populations in microbiome research.

## 5. The Gut Microbiome of South African Populations in Transition: From Paediatric Research to Emerging Adult Cohorts

South Africa ranks among the most genetically, ecologically, and culturally diverse countries in Sub-Saharan Africa, presenting a unique context for gut microbiome research [204]. Yet, despite this potential, local investigations have predominantly focused on paediatric populations, particularly infants and young children, leaving adult gut microbiome dynamics largely underexplored [33]. Early studies established foundational insights into diet–microbiome interactions. For instance, iron supplementation has been shown to influence gut inflammation and bacterial populations in children, highlighting the sensitivity of the developing microbiome to micronutrient interventions [205]. Fibre intake has also been linked to microbiota composition and food sensitisation in toddlers, emphasising diet-driven modulation of early immune development [206]. These observations were further expanded by research illustrating how HIV exposure, feeding practices, and delivery mode interact to shape neonatal bacterial profiles, demonstrating the complex interplay between early-life exposures and microbiome establishment [207].

Subsequent studies continued to investigate the combined effects of infection and nutritional status. Underweight children living with HIV in Cape Town exhibited altered microbial diversity, while stunting had been shown to influence gut microbiota, reinforcing the centrality of nutritional status in shaping microbial communities [208,209]. Disease-specific dysbiosis has also been characterised, with distinct microbial signatures identified in infants with respiratory and gastrointestinal conditions [210]. Later work integrated HIV status and micronutrient availability, examining gut microbiota in relation to HIV and iron levels, and considering clinical and environmental drivers of microbial profiles in young children [211,212]. Complementary investigations expanded analyses to maternal–infant microbiome interfaces through profiling of breastmilk, stool, and meconium, providing valuable insights into vertical microbial transmission [213]. Collectively, these studies illustrate that South African children’s gut microbiota are shaped by a constellation of factors including infectious status, micronutrient availability, feeding practices, and environmental exposures, with HIV and undernutrition consistently linked to reduced microbial diversity.

Despite these advances, the adult gut microbiome remains comparatively understudied in South Africa. Emerging research has begun to address urban–rural gradients and dietary transitions, yet the focus has largely been descriptive, emphasising bacterial composition without integrating functional interactions or cross-kingdom dynamics. While bacteria dominate the gut microbiome, understanding their role in isolation risks overlooking ecological and metabolic interactions that underpin health outcomes. Nevertheless, these studies signal an important shift toward lifespan-spanning microbiome research that considers environmental, dietary and cultural influences on gut health.

Multi-country studies have provided additional context for understanding African adult microbiomes. Analyses leveraging the Modeling the Epidemiologic Transition Study (METS) compared microbiota and cardiometabolic risk across Ghana, South Africa, Jamaica, and the United States [214]. Their findings demonstrated clear urbanisation- and lifestyle-associated gradients: rural African participants exhibited higher gut microbial diversity and greater abundance of fibre-degrading taxa such as *Prevotella*, correlating with lower cardiometabolic risk, whereas urbanised cohorts, particularly in the U.S., showed reduced diversity and dominance of taxa like *Bacteroides* and *Ruminococcus*, associated with obesity, insulin resistance, and hypertension. These results illustrate the interaction between environmental transitions, microbial ecology, and health outcomes, reinforcing the need to consider socio-geographic and lifestyle contexts when interpreting microbiome data [214].

Within South Africa, the Africa Wits-INDEPTH Partnership for Genomic Studies (AWI-Gen) has contributed to understanding adult gut microbiomes by integrating genetic, environmental, and microbial analyses [39]. Profiling of 170 HIV-negative women (119 rural Bushbuckridge, 51 urban Soweto) using 16S rRNA sequencing showed that geographic location explained more variation in microbial composition than BMI. Rural participants exhibited higher microbial diversity and greater abundance of *Prevotella*, *Vampirovibrio* (a *Melainabacterium*), and *Phascolarctobacterium*, whereas urban Soweto samples were enriched in Western-associated genera such as *Bacteroides*, *Bifidobacterium*, and *Barnesiella*. Interestingly, *Prevotella* abundance positively correlated with obesity in Bushbuckridge, suggesting that traditional markers of fibre-associated microbes may exhibit context-dependent relationships with health outcomes in transitional populations [39].

Building on these findings, Tamburini et al. (2022) [130] applied combined short-read Illumina and long-read Oxford Nanopore shotgun metagenomics to 169 South African adult women (118 rural, 51 urban), providing genome-resolved insights [130]. This approach revealed previously undescribed taxa, including *Treponema* and *Succinatimonas*, which were enriched in rural participants, reflecting metagenomics’ ability to capture under-characterised rural-enriched taxa. Both rural and urban cohorts displayed a transitional microbiome between Western and traditional profiles: rural samples had higher diversity and enrichment of VANISH (Volatile, Associated Negatively with Industrialized Societies of Humans) taxa such as *Treponema* and *Succinivibrio*, whereas urban samples were dominated by Western-associated genera such as *Bacteroides*, *Bifidobacterium* and crAssphage viruses. Urban samples also contained higher proportions of human DNA suggesting increased epithelial turnover, low-grade inflammation, or environmental stressors [130].

While metagenomic sequencing provided in-depth taxonomic and functional resolution, some taxa such as *Barnesiella* were under-detected relative to 16S rRNA sequencing [39], likely due to differences in analytical workflows, reference database completeness, sequencing coverage and inherent biases in genome repositories towards Western populations. This highlights the continued importance of complementary sequencing approaches to fully capture microbial diversity in African cohorts.

Beyond descriptive taxonomic profiling, a multi-omic framework was applied to investigate how urbanisation and diet influence the gut microbiome, virome, and metabolome in relation to CRC risk among middle-aged Xhosa adults [34]. By comparing a rural cohort from Zithulele Hospital in the Eastern Cape with an urban cohort from the densely populated Khayelitsha township in Cape Town, the study revealed the marked impact of lifestyle transitions on microbial ecology. Urban participants consumed significantly more energy (~3578 kcal/day vs. 2185 kcal/day) with higher intakes of fat and animal protein, which corresponded to reduced gut microbial diversity and a depletion of fibre-degrading taxa such as *Prevotella* and *Treponema*. In parallel, urban microbiomes were enriched for bile-tolerant and pro-inflammatory genera, including *Bacteroides*, *Alistipes*, *Bilophila*, *Fusobacteria*, and *Lachnoclostridium*, many previously implicated in CRC pathogenesis [34].

While urbanisation and diet were associated with gut microbiome compositional shifts, the study remains largely correlative. Reduced microbial diversity in urban participants included depletion of fibre-degrading taxa such as *Prevotella*, *Treponema*, and *Succinivibrio*, which often support SCFA production, mucosal integrity, and metabolic health [215]. Loss of these taxa under Westernised dietary patterns may impair SCFA-mediated barrier support and immune regulation, reflecting a reduction in functional resilience [216]. Concurrently, urban microbiomes were enriched for *Bacteroides*, *Alistipes*, *Bilophila*, and *Lachnoclostridium*, genera involved in bile acid transformation and sulphite metabolism, which can promote pro-inflammatory states, increase exposure to harmful metabolites, and elevate risk for nutrition-related chronic diseases, obesity, and type II diabetes [217].

Metabolomic analyses showed elevated faecal deoxycholic acid in urban participants, indicating a potential mechanistic link to CRC, while SCFA levels remained comparable between groups, which raises uncertainty about whether reduced microbial diversity directly leads to functional deficits [34]. The virome also shifted in the urban cohort, with increased abundance of phages associated with Bacteroidota, suggesting diet-driven restructuring of bacterium–phage interactions, although the health implications remain unclear. Additional microbial profiles from food and skin samples highlight the influence of environmental exposures and lifestyle, while also illustrating the difficulty of determining direct causal effects on gut microbial ecology [34]. Notably, *Fusobacterium* species may actively penetrate the epithelial barrier and modulate immune responses, potentially increasing CRC risk [218].

Patterns of microbial variation observed in these studies appear to be associated with urbanisation and dietary Westernisation [219]. Increased consumption of processed foods, food additives such as emulsifiers and preservatives, higher intake of dietary fat and animal protein, and reduced dietary fibre have been associated with systematic reductions in microbial diversity [220]. These dietary shifts promote the loss of fibre-degrading taxa and the enrichment of bile-tolerant and pro-inflammatory microbes [221]. The resulting changes in microbial composition alter key metabolic outputs, leading to reduced production of protective SCFAs, increased concentrations of secondary bile acids, and accumulation of metabolites associated with inflammation, metabolic dysfunction and heightened CRC risk [220,222].

While earlier work had previously demonstrated how geography and lifestyle transitions influence gut microbial diversity and health outcomes, Jorgensen et al. (2025) [223] extended this perspective through a cross-continental investigation of environmental exposures. Their study, nested within the Modeling the Epidemiologic Transition Study (METS), was designed to explore how industrialisation and urbanisation impact metabolic health across African-origin populations. The effect of toxic metal exposure on gut microbiota and metabolic disease risk in adults from Ghana, South Africa, Jamaica, Seychelles, and the USA was examined. In a subset of 178 participants, they analysed stool samples using 16S rRNA gene sequencing to profile gut bacterial communities and matched urine samples were tested for lead, arsenic, cadmium and mercury concentrations. Their findings revealed marked geographic differences in microbiome composition and metal exposure. Ghana and South Africa exhibited the highest microbial diversity, with dominant genera such as *Prevotella*, *Christensenella*, and *Clostridium*, while the USA cohort showed the lowest diversity, composed of *Bacteroides*, *Parabacteroides*, and *Haemophilus*. Interestingly, Ghana had the highest levels of lead (83%) and arsenic (80%), which correlated with increased abundance of *Prevotella*, *Alloprevotella* and *Clostridium*: taxa positively associated with obesity and type 2 diabetes mellitus (T2DM) [223]. Similarly, the South African participants displayed high microbial diversity and elevated levels of *Clostridium*, *Peptostreptococcales* and *Ruminococcus*, with both lead and mercury exposures contributing to compositional shifts linked to the risk of metabolic dysfunction [223].

Overall, lead and arsenic were reported to be associated with changes in gut microbial composition across the populations studied, including increases in taxa previously linked with disease and decreases in genera often considered beneficial [223]. Functional pathway predictions suggested enrichment of microbial processes related to metabolic stress. Lead and arsenic were associated with altered gut microbiota and enriched metabolic stress pathways in this cross-sectional cohort, but causality cannot be inferred from these data [223]. These findings indicate correlations rather than causal effects; nevertheless, they are consistent with the hypothesis that heavy-metal exposure may contribute to microbiome alterations that, in turn, could influence metabolic risk, with patterns that vary by geography and diet.

Expanding our understanding of geographic influences on the gut microbiome of African populations, Maghini et al. (2025) [36] undertook the AWI-Gen 2 Microbiome Project, which analysed stool samples from 1801 African women across six sites in Burkina Faso, Ghana, Kenya and South Africa using shotgun metagenomics. They found that geographic location, rather than urban or rural classification, was the primary determinant of gut microbiome composition [36]. Urban populations (e.g., Soweto) exhibited increased *Bifidobacterium* and *Bacteroides*, while rural populations had higher *Treponema*, *Cryptobacteroides*, and *Prevotella*. Notably, *Treponema succinifaciens* presence was associated with low antibiotic use and high dietary fibre, suggesting environmental and nutritional factors shape its persistence [215]. The study also uncovered 1005 novel bacterial genomes and over 40,000 new viral genomes, including crAssphage. An HIV-associated microbiome signature was detected, including taxa such as *Dysosmobacter welbionis* and *Enterocloster* spp., with lower microbial diversity observed among people living with HIV (PLWH) [36]. These findings emphasise the importance of local context and lifestyle factors rather than simplistic urban–rural classifications in shaping the gut microbiome of African populations.

Drawing from all these findings, the gut microbiome of South African populations, across both paediatric and adult populations, displays a highly dynamic and transitional profile shaped by urbanisation, diet, environment, infection and nutritional status. Rural populations consistently show enrichment of traditional and fibre-degrading taxa such as *Prevotella*, *Treponema*, *Succinatimonas*, *Vampirovibrio* (Melainabacteria), *Phascolarctobacterium* and *Cryptobacteroides* [34,36,39,130,214,223]. In contrast, urban cohorts demonstrate higher abundances of genera associated with Westernised lifestyles, including *Bacteroides*, *Bifidobacterium*, *Barnesiella*, *Lachnoclostridium*, *Alistipes*, *Bilophila*, *Fusobacteria*, *Clostridium*, *Peptostreptococcales*, *Ruminococcus*, *Dysosmobacter welbionis*, and *Enterocloster* spp [34,36,39,130,214,223]. Viral populations also differ notably; while multiple studies report greater crAssphage prevalence in urban groups, Ramaboli et al. (2024) [34] identified higher crAssphage abundance in rural individuals, suggesting that local dietary patterns, environmental exposures or methodological factors may influence these trends [34]. These inconsistencies foreground an urgent need for standardised and comprehensive virome profiling within African settings. These rural–urban contrasts are further summarised in Table 2, which outlines differences in microbial diversity, compositional shifts, and inflammation-related markers. Furthermore, environmental toxicants such as lead and mercury have been linked to shifts favouring taxa implicated in metabolic disease risk [223], while HIV infection is associated with decreased microbial diversity and distinct compositional signatures [36]. Overall, these complex microbial patterns resist simple urban–rural classifications, instead revealing a rich and evolving landscape shaped by intertwined lifestyle habits, environmental exposures and health conditions. This intricate interplay highlights the urgent need for comprehensive, multi-domain microbiome research in South Africa that accounts for local context, enabling a deeper understanding of microbial ecology and its critical role in health outcomes.

## 6. Conclusions

The gut microbiome of African populations represents a critical yet underexplored frontier in understanding the continent’s evolving health landscape. In countries like South Africa, rapid urbanisation and dietary transitions are reshaping microbial communities in ways that may drive chronic disease. Urban communities increasingly consume energy-dense, low-fibre diets driven by globalisation and socioeconomic change, leading to a measurable loss of beneficial, diversity-promoting taxa such as *Faecalibacterium prausnitzii* and *Roseburia*. Concurrently, these shifts foster the expansion of potentially pro-inflammatory microbes, including *Escherichia coli* and *Collinsella*, with clear associations to rising rates of obesity, CRC and systemic inflammation, especially in metropolitan centres like Johannesburg, Cape Town and Durban.

However, caution is warranted against broad generalisations across Africa. South Africa’s profound internal diversity spans from ethnicity, geography, dietary patterns to socioeconomic status, mirroring the continent’s complexity. Rural populations in regions such as the Eastern Cape and Bushbuckridge often retain microbiomes characterised by fibre-degrading genera like *Prevotella*, reflecting sustained traditional diets rich in plant fibre. These communities provide essential insights into pre-industrial microbial configurations and illustrate the potential to preserve microbial diversity through lifestyle choices.

To advance microbiome science and inform public health effectively, research must adopt a more nuanced and inclusive framework that encompasses the full spectrum of South African environments. Longitudinal and multi-omic approaches integrating genomic, dietary, environmental and detailed clinical data are critical to unravel how microbial communities mediate health outcomes, especially in the face of endemic conditions like HIV, tuberculosis, and childhood malnutrition. Such a comprehensive understanding is fundamental to designing targeted, culturally sensitive interventions. For example, urban health policies might focus on promoting fibre-rich diets, limiting ultra-processed foods and recognising microbial health as a pillar in chronic disease prevention. To fully realise its potential, the human gut microbiome must be studied comprehensively across diverse populations and contexts, ensuring that insights translate into health interventions tailored to the unique environmental, cultural and epidemiological realities of each global community.

## Figures and Tables

**Table 1:** A summary of typical abundance patterns, dominant genera, core functional roles, and associated disease outcomes across key microbial groups, integrating genera commonly observed in both Western and African settings.

Microbial Group	Typical Abundance	Dominant Genera	Core Functional Roles	Associated Disease Outcomes	Key References
Fungi (Mycobiome)	Low relative abundance; <0.1% of gut microbiota	*Candida*, *Saccharomyces*, *Pichia*, *Aspergillus*, *Cladosporium*	Immune modulation, SCFA interactions, cross-kingdom signalling	IBS, Crohn’s disease, leaky gut syndrome, inflammation	[111,114,119]
Viruses (Virome)	Highly individual-specific; thousands of contigs; bacteriophages dominate	crAssphage, Anelloviruses, Podoviridae, Siphoviridae	Bacterial population control, immune modulation, gene transfer	HIV-related immune suppression, IBD, early CRC markers	[34,128,130]
Archaea	Low diversity but stable presence; individual-specific	*Methanobrevibacter*, *Methanosphaera*, Methanomassiliicoccales	Methanogenesis, hydrogen scavenging, TMA reduction	IBD, metabolic disorders, possible cardiovascular effects	[122,143,149]
Protozoa and Helminths	Frequently excluded from gut microbiome datasets	*Blastocystis* (subtypes), others not often identified	Immune conditioning, microbial diversity regulation	Reduced vaccine efficacy, HIV/TB coinfection impact, context-dependent roles	[156,161,162]

**Table 2:** Comparison of gut microbiome features in rural vs. urban population: diversity, taxa shifts, dietary context, inflammatory associations and HIV status.

Feature	Rural Populations	Urban Populations	Relevant Studies
Microbial diversity	Higher alpha diversity reported in Bushbuckridge and Eastern Cape; *Prevotella*-rich profiles retained in less industrialised areas (e.g., Ghana, rural SA); diversity linked to traditional dietary practices	Lower microbial diversity observed in Soweto, Khayelitsha, and US cohorts; reduced beta diversity associated with lead/mercury exposure; microbial loss correlated with Westernised diets	[34,39,130,214,223]
Dominant fibre-degrading or VANISH taxa	Enriched *Prevotella*, *Treponema*, *Succinatimonas*, *Succinivibrio*, *Vampirovibrio*, *Phascolarctobacterium*, and *Cryptobacteroides*; associated with high-fibre, plant-based diets	Lower abundance or loss of these taxa; urban diets marked by higher energy, fat, and animal protein intake (e.g., ~3578 kcal/day in Khayelitsha) contributed to depletion of *Prevotella* and *Treponema*	[34,36,39,130]
Western-associated or pro-inflammatory taxa	Largely absent or less prevalent; lower abundance of bile-tolerant and inflammation-linked genera	Enrichment of *Bacteroides*, *Bifidobacterium*, *Barnesiella*, *Alistipes*, *Bilophila*, *Lachnoclostridium*, *Haemophilus*, crAssphage; associated with CRC and metabolic risk	[34,36,39,130,214,223]
Dietary patterns	Traditional, plant-based diets rich in fibre; low antibiotic use (e.g., *Treponema succinifaciens* persistence); lower energy intake (~2185 kcal/day); protective metabolic profile	Energy-dense diets with higher fat and animal protein intake in urban areas (e.g., Khayelitsha); associated with pro-inflammatory shifts in microbiota and bile acid metabolism	[34,36]
Metabolic/inflammatory risk markers	Lower levels of CRC- and T2DM-associated taxa; microbiota associated with favourable metabolic outcomes; higher *Prevotella* in Bushbuckridge linked to low-grade inflammation but unclear risk	Higher BMI, T2DM risk, and faecal deoxycholic acid (a CRC-associated metabolite) in urban individuals; toxicant exposure (lead, mercury) linked to shifts in pro-inflammatory taxa and metabolic stress	[34,39,214,223]
Inflammation and gut barrier integrity	No evidence of compromised gut barrier; overall diversity may confer protection	Soweto samples had elevated human DNA in stool, suggesting epithelial cell turnover, barrier disruption, or low-grade inflammation; HIV-associated microbiome showed reduced diversity	[36,130]
Environmental exposures	Less exposure was mentioned in rural settings	Urban samples (South Africa, USA) showed higher lead and mercury levels; associated with lower diversity and enrichment of *Clostridium*, *Peptostreptococcales*, and *Ruminococcus*	[214,223]
HIV statuses/associations	No rural cohorts were reported to have individuals living with HIV; Oduaran et al. (2020) [39] and Tamburini et al. (2022) [130] used individuals who tested negative for HIV; Maghini et al. (2025) [36] noted HIV-associated taxa in some rural samples (*Dysosmobacter welbionis*)	HIV-associated microbial signature (*Dysosmobacter welbionis*, *Enterocloster* spp.) with reduced diversity reported in urban Soweto; only Maghini et al. (2025) [36] included PLWH in their sample	[36,39,130]

## Data Availability

No new data were created or analyzed in this study. Data sharing is not applicable to this article.

## References

[1] Sasso J.M., Ammar R.M., Tenchov R., Lemmel S., Kelber O., Grieswelle M., Zhou Q.A. (2023). Gut microbiome–brain alliance: A landscape view into mental and gastrointestinal health and disorders. ACS Chem. Neurosci..

[2] Schmidt H.H.H.W. (2022). Outnumbered. The End of Medicine as We Know It—And Why Your Health Has a Future.

[3] Bhatt B., Patel K., Lee C.N., Moochhala S. (2024). The Microbial Blueprint: The Impact of Your Gut on Your Well-Being.

[4] Dhanaraju R., Rao D.N. (2022). The human microbiome: An acquired organ?. Resonance.

[5] Imran M., Ahmad B. (2024). Microbiome and its impact on human health: Microbiome in various body organs and its association with human health and disease. The Microbiome and Cancer: Understanding the Role of Microorganisms in Tumor Development and Treatment.

[6] Rothschild D., Weissbrod O., Barkan E., Kurilshikov A., Korem T., Zeevi D., Costea P.I., Godneva A., Kalka I.N., Bar N. (2018). Environment dominates over host genetics in shaping human gut microbiota. Nature.

[7] Hussain M.S., Bahl G., Mishra R., Bhat A.A., Thapa R., Siddiqui R., Sharma R., Kulshrestha R., Patel N., Gupta G. (2025). Introduction to Microbiome. Gut Microbiome and Environmental Toxicants.

[8] Qin Y., Havulinna A.S., Liu Y., Jousilahti P., Ritchie S.C., Tokolyi A., Sanders J.G., Valsta L., Brożyńska M., Zhu Q. (2022). Combined effects of host genetics and diet on human gut microbiota and incident disease in a single population cohort. Nat. Genet..

[9] Robitaille S., Simmons E.L., Verster A.J., McClure E.A., Royce D.B., Trus E., Swartz K., Schultz D., Nadell C.D., Ross B.D. (2023). Community composition and the environment modulate the population dynamics of type VI secretion in human gut bacteria. Nat. Ecol. Evol..

[10] Milani C., Duranti S., Bottacini F., Casey E., Turroni F., Mahony J., Belzer C., Delgado Palacio S., Arboleya Montes S., Mancabelli L. (2017). The first microbial colonizers of the human gut: Composition, activities, and health implications of the infant gut microbiota. Microbiol. Mol. Biol. Rev..

[11] Costea P.I., Coelho L.P., Sunagawa S., Munch R., Huerta-Cepas J., Forslund K., Hildebrand F., Kushugulova A., Zeller G., Bork P. (2017). Subspecies in the global human gut microbiome. Mol. Syst. Biol..

[12] Camarillo-Guerrero L.F., Almeida A., Rangel-Pineros G., Finn R.D., Lawley T.D. (2021). Massive expansion of human gut bacteriophage diversity. Cell.

[13] Anderson B.D., Bisanz J.E. (2023). Challenges and opportunities of strain diversity in gut microbiome research. Front. Microbiol..

[14] Zeng S., Patangia D., Almeida A., Zhou Z., Mu D., Paul Ross R., Stanton C., Wang S. (2022). A compendium of 32,277 metagenome-assembled genomes and over 80 million genes from the early-life human gut microbiome. Nat. Commun..

[15] Hampton T. (2018). Gut microbes may shape response to cancer immunotherapy. JAMA.

[16] Song M., Chan A.T., Sun J. (2020). Influence of the gut microbiome, diet, and environment on risk of colorectal cancer. Gastroenterology.

[17] Wernroth M.L., Peura S., Hedman A.M., Hetty S., Vicenzi S., Kennedy B., Fall K., Svennblad B., Andolf E., Pershagen G. (2022). Development of gut microbiota during the first 2 years of life. Sci. Rep..

[18] Gilbert M.S., Ijssennagger N., Kies A.K., van Mil S.W. (2018). Protein fermentation in the gut; implications for intestinal dysfunction in humans, pigs, and poultry. Am. J. Physiol.-Gastrointest. Liver Physiol..

[19] Pham V.T., Fehlbaum S., Seifert N., Richard N., Bruins M.J., Sybesma W., Rehman A., Steinert R.E. (2021). Effects of colon-targeted vitamins on the composition and metabolic activity of the human gut microbiome—A pilot study. Gut Microbes.

[20] Zhan Q., Wang R., Thakur K., Feng J.Y., Zhu Y.Y., Zhang J.G., Wei Z.J. (2024). Unveiling of dietary and gut-microbiota derived B vitamins: Metabolism patterns and their synergistic functions in gut-brain homeostasis. Crit. Rev. Food Sci. Nutr..

[21] Liu Y., Wang Y., Ni Y., Cheung C.K.Y., Lam K.S.L., Wang Y., Xia Z., Ye D., Guo J., Tse M.A. (2020). Gut Microbiome Fermentation Determines the Efficacy of Exercise for Diabetes Prevention. Cell Metab..

[22] Martinez J.E., Kahana D.D., Ghuman S., Wilson H.P., Wilson J., Kim S.C., Lagishetty V., Jacobs J.P., Sinha-Hikim A.P., Friedman T.C. (2021). Unhealthy lifestyle and gut dysbiosis: A better understanding of the effects of poor diet and nicotine on the intestinal microbiome. Front. Endocrinol..

[23] Aljumaah M.R., Bhatia U., Roach J., Gunstad J., Azcarate Peril M.A. (2022). The gut microbiome, mild cognitive impairment, and probiotics: A randomized clinical trial in middle aged and older adults. Clin. Nutr..

[24] Ni Y., Qian L., Siliceo S.L., Long X., Nychas E., Liu Y., Ismaiah M.J., Leung H., Zhang L., Gao Q. (2023). Resistant starch decreases intrahepatic triglycerides in patients with NAFLD via gut microbiome alterations. Cell Metab..

[25] Procházková N., Venlet N., Hansen M.L., Lieberoth C.B., Dragsted L.O., Bahl M.I., Licht T.R., Kleerebezem M., Lauritzen L., Roager H.M. (2023). Effects of a wholegrain-rich diet on markers of colonic fermentation and bowel function and their associations with the gut microbiome: A randomised controlled cross-over trial. Front. Nutr..

[26] Wei S., Bahl M.I., Baunwall S.M.D., Hvas C.L., Licht T.R. (2021). Determining gut microbial dysbiosis: A review of applied indexes for assessment of intestinal microbiota imbalances. Appl. Environ. Microbiol..

[27] de Oliveira G.L.V., Cardoso C.R.D.B., Taneja V., Fasano A. (2021). Intestinal dysbiosis in inflammatory diseases. Front. Immunol..

[28] Chen L., Liu B., Ren L., Du H., Fei C., Qian C., Li B., Zhang R., Liu H., Li Z. (2023). High-fiber diet ameliorates gut microbiota, serum metabolism and emotional mood in type 2 diabetes patients. Front. Cell. Infect. Microbiol..

[29] Brüssow H. (2020). Problems with the concept of gut microbiota dysbiosis. Microb. Biotechnol..

[30] Nawaz A., Zafar S., Shahzadi M., Sharif M., Saeeda U.H., Khalid N.A., Khan S. (2024). Correlation between gut microbiota and chronic metabolic diseases. Role of Flavonoids in Chronic Metabolic Diseases: From Bench to Clinic.

[31] de Vos W.M., Tilg H., Van Hul M., Cani P.D. (2022). Gut microbiome and health: Mechanistic insights. Gut.

[32] Afzaal M., Saeed F., Shah Y.A., Hussain M., Rabail R., Socol C.T., Hassoun A., Pateiro M., Lorenzo J.M., Rusu A.V. (2022). Human gut microbiota in health and disease: Unveiling the relationship. Front. Microbiol..

[33] Littlejohn P.T., Glover J.S. (2023). Ethical gut microbiota research in Africa. Nat. Microbiol..

[34] Ramaboli M.C., Ocvirk S., Khan Mirzaei M., Eberhart B.L., Valdivia-Garcia M., Metwaly A., Neuhaus K., Barker G., Ru J., Nesengani L.T. (2024). Diet changes due to urbanization in South Africa are linked to microbiome and metabolome signatures of Westernization and colorectal cancer. Nat. Commun..

[35] Moyo G.T., Tepekule B., Katsidzira L., Blaser M.J., Metcalf C.J.E. (2025). Getting ahead of human-associated microbial decline in Africa: The urgency of sampling in light of epidemiological transition. Trends Microbiol..

[36] Maghini D.G., Oduaran O.H., Olubayo L.A.I., Cook J.A., Smyth N., Mathema T., Belger C.W., Agongo G., Boua P.R., Choma S.S. (2025). Expanding the human gut microbiome atlas of Africa. Nature.

[37] Brewster R., Tamburini F.B., Asiimwe E., Oduaran O., Hazelhurst S., Bhatt A.S. (2019). Surveying gut microbiome research in Africans: Toward improved diversity and representation. Trends Microbiol..

[38] Pasolli E., Asnicar F., Manara S., Zolfo M., Karcher N., Armanini F., Beghini F., Manghi P., Tett A., Ghensi P. (2019). Extensive unexplored human microbiome diversity revealed by over 150,000 genomes from metagenomes spanning age, geography, and lifestyle. Cell.

[39] Oduaran O.H., Tamburini F.B., Sahibdeen V., Brewster R., Gómez-Olivé F.X., Kahn K., Norris S.A., Tollman S.M., Twine R., Wade A.N. (2020). Gut microbiome profiling of a rural and urban South African cohort reveals biomarkers of a population in lifestyle transition. BMC Microbiol..

[40] Fontaine F., Turjeman S., Callens K., Koren O. (2023). The intersection of undernutrition, microbiome, and child development in the first years of life. Nat. Commun..

[41] Gomez A., Petrzelkova K.J., Burns M.B., Yeoman C.J., Amato K.R., Vlckova K., Modry D., Todd A., Jost Robinson C.A., Remis M.J. (2016). Gut microbiome of coexisting BaAka Pygmies and Bantu reflects gradients of traditional subsistence patterns. Cell Rep..

[42] de Filippo C., Di Paola M., Ramazzotti M., Albanese D., Pieraccini G., Banci E., Miglietta F., Cavalieri D., Lionetti P. (2017). Diet, environments, and gut microbiota. A preliminary investigation in children living in rural and urban Burkina Faso and Italy. Front. Microbiol..

[43] Ayeni K.I., Berry D., Wisgrill L., Warth B., Ezekiel C.N. (2022). Early-life chemical exposome and gut microbiome development: African research perspectives within a global environmental health context. Trends Microbiol..

[44] Truter M. (2021). The taxonomic diversity of the Ju|’hoansi hunter-gatherer intestinal microbiome in Tsumkwe, Namibia. Ph.D. Thesis.

[45] Al Dhaheri A.S., Alkhatib D.H., Feehan J., Cheikh Ismail L., Apostolopoulos V., Stojanovska L. (2024). The effect of therapeutic doses of culinary spices in metabolic syndrome: A randomized controlled trial. Nutrients.

[46] Taheri Soodejani M. (2024). Non-communicable diseases in the world over the past century: A secondary data analysis. Front. Public Health.

[47] Gouda H.N., Charlson F., Sorsdahl K., Ahmadzada S., Ferrari A.J., Erskine H., Leung J., Santamauro D., Lund C., Aminde L.N. (2019). Burden of non-communicable diseases in sub-Saharan Africa, 1990–2017: Results from the Global Burden of Disease Study 2017. Lancet Glob. Health.

[48] Wong E.B., Olivier S., Gunda R., Koole O., Surujdeen A., Gareta D., Munatsi D., Modise T.H., Dreyer J., Nxumalo S. (2021). Convergence of infectious and non-communicable disease epidemics in rural South Africa: A cross-sectional, population-based multimorbidity study. Lancet Glob. Health.

[49] Achoki T., Sartorius B., Watkins D., Glenn S.D., Kengne A.P., Oni T., Wiysonge C.S., Walker A., Adetokunboh O.O., Babalola T.K. (2022). Health trends, inequalities and opportunities in South Africa’s provinces, 1990–2019: Findings from the Global Burden of Disease 2019 Study. J. Epidemiol. Community Health.

[50] Johnson L.F., Kassanjee R., Folb N., Bennett S., Boulle A., Levitt N.S., Curran R., Bobrow K., Roomaney R.A., Bachmann M.O. (2024). A model-based approach to estimating the prevalence of disease combinations in South Africa. BMJ Glob. Health.

[51] Modjadji P. (2021). Communicable and non-communicable diseases coexisting in South Africa. Lancet Glob. Health.

[52] Roomaney R.A., Van Wyk B., Cois A., Pillay van-Wyk V. (2023). Multimorbidity patterns in South Africa: A latent class analysis. Front. Public Health.

[53] Owino V.O. (2019). Challenges and opportunities to tackle the rising prevalence of diet-related non-communicable diseases in Africa. Proc. Nutr. Soc..

[54] Ciccacci F., Welu B., Ndoi H., Mosconi C., De Santo C., Carestia M., Altan A.M.D., Murungi J., Muthuri K., Cicala M. (2024). Exploring diseases burden in HIV population: Results from the CHAO (Comorbidities in HIV/AIDS outpatients) cross-sectional study in Kenya. Glob. Epidemiol..

[55] de Bruin S., Dengerink J., van Vliet J. (2021). Urbanisation as driver of food system transformation and opportunities for rural livelihoods. Food Secur..

[56] Nasreddine L., Hamdan A.L., Sataloff R.T., Hawkshaw M.J. (2022). Urbanization, transition in diet and voice. Traits of Civilization and Voice Disorders.

[57] Alam M.R., Begum M., Sharmin R., Naser A.Z.M., Rahman M.M., Hossain M.A., Tanveer S.K.M., Parves M.M., Ahmed E., Akter T. (2024). Obesity in Southeast Asia: An emerging health concern. Sch. J. Appl. Med. Sci..

[58] Mostafa I., Lamiya U.H., Rasul M.G., Naila N.N., Fahim S.M., Hasan S.M.T., Barratt M.J., Gordon J.I., Ahmed T. (2024). Development and acceptability of shelf-stable microbiota directed complementary food formulations. Food Nutr. Bull..

[59] Pheeha S.M., Tamuzi J.L., Chale-Matsau B., Manda S., Nyasulu P.S. (2023). A scoping review evaluating the current state of gut microbiota research in Africa. Microorganisms.

[60] Allali I., Abotsi R.E., Tow L.A., Thabane L., Zar H.J., Mulder N.M., Nicol M.P. (2021). Human microbiota research in Africa: A systematic review reveals gaps and priorities for future research. Microbiome.

[61] Joos R., Boucher K., Lavelle A., Arumugam M., Blaser M.J., Claesson M.J., Clarke G., Cotter P.D., De Sordi L., Dominguez-Bello M.G. (2025). Examining the healthy human microbiome concept. Nat. Rev. Microbiol..

[62] Nienaber-Rousseau C. (2025). Understanding and applying gene–environment interactions: A guide for nutrition professionals with an emphasis on integration in African research settings. Nutr. Rev..

[63] Nkera-Gutabara C.K., Kerr R., Scholefield J., Hazelhurst S., Naidoo J. (2022). Microbiomics: The next pillar of precision medicine and its role in African healthcare. Front. Genet..

[64] Kouidhi S., Oduaran O.H. (2024). Strengthening the foundation of African microbiome research: Strategies for standardized data collection. Nat. Rev. Gastroenterol. Hepatol..

[65] Vuik F.E.R., Dicksved J., Lam S.Y., Fuhler G.M., van der Laan L.J.W., van de Winkel A., Konstantinov S.R., Spaander M.C.W., Peppelenbosch M.P., Engstrand L. (2019). Composition of the mucosa-associated microbiota along the entire gastrointestinal tract of human individuals. United Eur. Gastroenterol. J..

[66] Banerjee P., Adhikary K., Chatterjee A., Sarkar R., Bagchi D., Ghosh N., Das A. (2022). Digestion and gut microbiome. Nutrition and Functional Foods in Boosting Digestion, Metabolism and Immune Health.

[67] Passos M.D.C.F., Moraes-Filho J.P. (2017). Intestinal microbiota in digestive diseases. Arq. Gastroenterol..

[68] Selma-Royo M., Calatayud Arroyo M., García-Mantrana I., Parra-Llorca A., Escuriet R., Martínez-Costa C., Collado M.C. (2020). Perinatal environment shapes microbiota colonization and infant growth: Impact on host response and intestinal function. Microbiome.

[69] Hillman E.T., Lu H., Yao T., Nakatsu C.H. (2017). Microbial ecology along the gastrointestinal tract. Microbes Environ..

[70] Quigley E.M. (2013). Gut bacteria in health and disease. Gastroenterol. Hepatol..

[71] Suárez-Martínez C., Santaella-Pascual M., Yagüe-Guirao G., Martínez-Graciá C. (2023). Infant gut microbiota colonization: Influence of prenatal and postnatal factors, focusing on diet. Front. Microbiol..

[72] Shkoporov A.N., Turkington C.J., Hill C. (2022). Mutualistic interplay between bacteriophages and bacteria in the human gut. Nat. Rev. Microbiol..

[73] Suzuki T.A., Fitzstevens J.L., Schmidt V.T., Enav H., Huus K.E., Mbong Ngwese M., Grießhammer A., Pfleiderer A., Adegbite B.R., Zinsou J.F. (2022). Codiversification of gut microbiota with humans. Science.

[74] Loo W.T., Dudaniec R.Y., Kleindorfer S., Cavanaugh C.M. (2019). An inter-island comparison of Darwin’s finches reveals the impact of habitat, host phylogeny, and island on the gut microbiome. PLoS ONE.

[75] Kijner S., Kolodny O., Yassour M. (2022). Human milk oligosaccharides and the infant gut microbiome from an eco-evolutionary perspective. Curr. Opin. Microbiol..

[76] Korpela K. (2021). Impact of delivery mode on infant gut microbiota. Ann. Nutr. Metab..

[77] Raspini B., Vacca M., Porri D., De Giuseppe R., Calabrese F.M., Chieppa M., Liso M., Cerbo R.M., Civardi E., Garofoli F. (2021). Early life microbiota colonization at six months of age: A transitional time point. Front. Cell. Infect. Microbiol..

[78] Shenhav L., Fehr K., Reyna M.E., Petersen C., Dai D.L., Dai R., Breton V., Rossi L., Smieja M., Simons E. (2024). Microbial colonization programs are structured by breastfeeding and guide healthy respiratory development. Cell.

[79] Wang X.A., Li J.P., Lee M.S., Yang S.F., Chang Y.S., Chen L., Li C.W., Chao Y.H. (2024). A common trajectory of gut microbiome development during the first month in healthy neonates with limited inter-individual environmental variations. Sci. Rep..

[80] Naspolini N.F., Natividade A.P., Asmus C.I.F., Moreira J.C., Dominguez-Bello M.G., Meyer A. (2025). Early-life gut microbiome is associated with behavioral disorders in the Rio birth cohort. Sci. Rep..

[81] Lloyd-Price J., Mahurkar A., Rahnavard G., Crabtree J., Orvis J., Hall A.B., Brady A., Creasy H.H., McCracken C., Giglio M.G. (2017). Strains, functions and dynamics in the expanded Human Microbiome Project. Nature.

[82] Mitsuoka T. (1992). Intestinal flora and aging. Nutr. Rev. Wash..

[83] Woodmansey E.J. (2007). Intestinal bacteria and ageing. J. Appl. Microbiol..

[84] Wastyk H.C., Fragiadakis G.K., Perelman D., Dahan D., Merrill B.D., Yu F.B., Topf M., Gonzalez C.G., Van Treuren W., Han S. (2021). Gut-microbiota-targeted diets modulate human immune status. Cell.

[85] Li H., Zhang L., Li J., Wu Q., Qian L., He J., Ni Y., Kovatcheva-Datchary P., Yuan R., Liu S. (2024). Resistant starch intake facilitates weight loss in humans by reshaping the gut microbiota. Nat. Metab..

[86] LeMay-Nedjelski L., Butcher J., Ley S.H., Asbury M.R., Hanley A.J., Kiss A., Unger S., Copeland J.K., Wang P.W., Zinman B. (2020). Examining the relationship between maternal body size, gestational glucose tolerance status, mode of delivery and ethnicity on human milk microbiota at three months post-partum. BMC Microbiol..

[87] McCann S.E., Hullar M.A.J., Tritchler D.L., Cortes-Gomez E., Yao S., Davis W., O’Connor T., Erwin D., Thompson L.U., Yan L. (2021). Enterolignan production in a flaxseed intervention study in postmenopausal US women of African ancestry and European ancestry. Nutrients.

[88] Deschasaux M., Bouter K.E., Prodan A., Levin E., Groen A.K., Herrema H., Tremaroli V., Bakker G.J., Attaye I., Pinto-Sietsma S.J. (2018). Depicting the composition of gut microbiota in a population with varied ethnic origins but shared geography. Nat. Med..

[89] Dwiyanto J., Hussain M.H., Reidpath D., Ong K.S., Qasim A., Lee S.W.H., Lee S.M., Foo S.C., Chong C.W., Rahman S. (2021). Ethnicity influences the gut microbiota of individuals sharing a geographical location: A cross-sectional study from a middle-income country. Sci. Rep..

[90] Bosch J.A., Nieuwdorp M., Zwinderman A.H., Deschasaux M., Radjabzadeh D., Kraaij R., Davids M., de Rooij S.R., Lok A. (2022). The gut microbiota and depressive symptoms across ethnic groups. Nat. Commun..

[91] Niccolai E., Di Gloria L., Trolese M.C., Fabbrizio P., Baldi S., Nannini G., Margotta C., Nastasi C., Ramazzotti M., Bartolucci G. (2024). Host genetics and gut microbiota influence lipid metabolism and inflammation: Potential implications for ALS pathophysiology in SOD1^G93A^ mice. Acta Neuropathol. Commun..

[92] Benson A.K., Kelly S.A., Legge R., Ma F., Low S.J., Kim J., Zhang M., Oh P.L., Nehrenberg D., Hua K. (2010). Individuality in gut microbiota composition is a complex polygenic trait shaped by multiple environmental and host genetic factors. Proc. Natl. Acad. Sci. USA.

[93] Zhernakova D.V., Wang D., Liu L., Andreu-Sánchez S., Zhang Y., Ruiz-Moreno A.J., Peng H., Plomp N., Del Castillo-Izquierdo Á., Gacesa R. (2024). Host genetic regulation of human gut microbial structural variation. Nature.

[94] Robinson J.M., Breed M.F. (2025). Beyond microbial exposure and colonization: Multisensory shaping of the gut microbiome. mSystems.

[95] Jeong S. (2021). Factors influencing development of the infant microbiota: From prenatal period to early infancy. Clin. Exp. Pediatr..

[96] Mogoş G.F.R., Manciulea M., Enache R.M., Pavelescu L.A., Popescu O.A., Cretoiu S.M., Marinescu I. (2025). Intestinal microbiota in early life: Latest findings regarding the role of probiotics as a treatment approach for dysbiosis. Nutrients.

[97] Iliev I.D., Ananthakrishnan A.N., Guo C.J. (2025). Microbiota in inflammatory bowel disease: Mechanisms of disease and therapeutic opportunities. Nat. Rev. Microbiol..

[98] Geng J., Ni Q., Sun W., Li L., Feng X. (2022). The links between gut microbiota and obesity and obesity related diseases. Biomed. Pharmacother..

[99] Yang H., Qu Y., Gao Y., Sun S., Wu R., Wu J. (2022). Research progress on the correlation between the intestinal microbiota and food allergy. Foods.

[100] Shen Y., Fan N., Ma S.X., Cheng X., Yang X., Wang G. (2025). Gut microbiota dysbiosis: Pathogenesis, diseases, prevention, and therapy. MedComm.

[101] Liu L., Wang H., Chen X., Zhang Y., Zhang H., Xie P. (2023). Gut microbiota and its metabolites in depression: From pathogenesis to treatment. eBioMedicine.

[102] Pérez J.C. (2021). Fungi of the human gut microbiota: Roles and significance. Int. J. Med. Microbiol..

[103] Limon J.J., Skalski J.H., Underhill D.M. (2017). Commensal fungi in health and disease. Cell Host Microbe.

[104] Richard M.L., Sokol H. (2019). The gut mycobiota: Insights into analysis, environmental interactions and role in gastrointestinal diseases. Nat. Rev. Gastroenterol. Hepatol..

[105] Moissl-Eichinger C., Pausan M., Taffner J., Berg G., Bang C., Schmitz R.A. (2018). Archaea are interactive components of complex microbiomes. Trends Microbiol..

[106] Shkoporov A.N., Hill C. (2019). Bacteriophages of the human gut: The “known unknown” of the microbiome. Cell Host Microbe.

[107] Gregory A.C., Zablocki O., Zayed A.A., Howell A., Bolduc B., Sullivan M.B. (2020). The gut virome database reveals age-dependent patterns of virome diversity in the human gut. Cell Host Microbe.

[108] Huang H., Wang Q., Yang Y., Zhong W., He F., Li J. (2024). The mycobiome as integral part of the gut microbiome: Crucial role of symbiotic fungi in health and disease. Gut Microbes.

[109] Nash A.K., Auchtung T.A., Wong M.C., Smith D.P., Gesell J.R., Ross M.C., Stewart C.J., Metcalf G.A., Muzny D.M., Gibbs R.A. (2017). The gut mycobiome of the Human Microbiome Project healthy cohort. Microbiome.

[110] Witherden E.A., Shoaie S., Hall R.A., Moyes D.L. (2017). The human mucosal mycobiome and fungal community interactions. J. Fungi.

[111] Hallen-Adams H.E., Suhr M.J. (2017). Fungi in the healthy human gastrointestinal tract. Virulence.

[112] Wu X., Xia Y., He F., Zhu C., Ren W. (2021). Intestinal mycobiota in health and diseases: From a disrupted equilibrium to clinical opportunities. Microbiome.

[113] Maas E., Penders J., Venema K. (2023). Fungal-bacterial interactions in the human gut of healthy individuals. J. Fungi.

[114] Kapitan M., Niemiec M.J., Steimle A., Frick J.S., Jacobsen I.D. (2019). Fungi as part of the microbiota and interactions with intestinal bacteria. Fungal Physiology and Immunopathogenesis.

[115] Liu A., Gao W.-Q., Zhu Y., Hou X., Chu H. (2022). Gut non-bacterial microbiota: Emerging link to irritable bowel syndrome. Toxins.

[116] Rashed A.A., Mahmoud M.M., Abd El-Rahman G.I. (2022). Gut microbiota alterations in irritable bowel syndrome: A systematic review. Arab J. Gastroenterol..

[117] Haak B.W., Lankelma J.M., Hugenholtz F., Belzer C., de Vos W.M., Wiersinga W.J., Nieuwdorp M. (2021). Longitudinal impact of antimicrobial therapy on the gut microbiome in critically ill patients. J. Antimicrob. Chemother..

[118] Nel Van Zyl K., Whitelaw A.C., Hesseling A.C., Seddon J.A., Demers A.M., Newton-Foot M. (2022). Fungal diversity in the gut microbiome of young South African children. BMC Microbiol..

[119] Kabwe M.H., Vikram S., Mulaudzi K., Jansson J.K., Makhalanyane T.P. (2020). The gut mycobiota of rural and urban individuals is shaped by geography. BMC Microbiol..

[120] Patnaik S., Durairajan S.S.K., Singh A.K., Krishnamoorthi S., Iyaswamy A., Mandavi S.P., Jeewon R., Williams L.L. (2024). Role of *Candida* species in pathogenesis, immune regulation, and prognostic tools for managing ulcerative colitis and Crohn’s disease. World J. Gastroenterol..

[121] Jawhara S. (2023). Healthy diet and lifestyle improve the gut microbiota and help combat fungal infection. Microorganisms.

[122] Brown E.M., Allen-Vercoe E. (2011). Phage-host interactions in the gut: Influence of temperate bacteriophages on the gut microbiome. J. Appl. Microbiol..

[123] Rowan-Nash A.D., Korry B.J., Mylonakis E., Belenky P. (2019). Cross-domain and viral interactions in the microbiome. Microbiol. Mol. Biol. Rev..

[124] Lathakumari R.H., Vajravelu L.K., Gopinathan A., Vimala P.B., Panneerselvam V., Ravi S.S.S., Thulukanam J. (2025). The gut virome and human health: From diversity to personalized medicine. Eng. Microbiol..

[125] Hetta H.F., Ahmed R., Ramadan Y.N., Fathy H., Khorshid M., Mabrouk M.M., Hashem M. (2025). Gut virome: New key players in the pathogenesis of inflammatory bowel disease. World J. Methodol..

[126] Wu Y., Cheng R., Lin H., Li L., Jia Y., Philips A., Zuo T., Zhang H. (2025). Gut virome and its implications in the pathogenesis and therapeutics of inflammatory bowel disease. BMC Med..

[127] Gavkare A.M., Nanaware N.L., Sonar M.N., Dhotre S.V., Mumbre S.S., Nagoba B.S. (2025). Gut microbiome and viral infections: A hidden nexus for immune protection. World J. Virol..

[128] Boukadida C., Peralta-Prado A., Chávez-Torres M., Romero-Mora K., Rincon-Rubio A., Ávila-Ríos S., Garrido-Rodríguez D., Reyes-Terán G., Pinto-Cardoso S. (2024). Alterations of the gut microbiome in HIV infection highlight human anelloviruses as potential predictors of immune recovery. Microbiome.

[129] Monaco C.L., Gootenberg D.B., Zhao G., Handley S.A., Ghebremichael M.S., Lim E.S., Lankowski A., Baldridge M.T., Wilen C.B., Flagg M. (2016). Altered virome and bacterial microbiome in human immunodeficiency virus-associated acquired immunodeficiency syndrome. Cell Host Microbe.

[130] Tamburini F.B., Maghini D., Oduaran O.H., Brewster R., Hulley M.R., Sahibdeen V., Norris S.A., Tollman S., Kahn K., Wagner R.G. (2022). Short-and long-read metagenomics of urban and rural South African gut microbiomes reveal a transitional composition and undescribed taxa. Nat. Commun..

[131] Babkin I.V., Fedorets V.A., Tikunov A.Y., Baykov I.K., Panina E.A., Tikunova N.V. (2025). Zeta CrAss-like Phages, a Separate Phage Family Using a Variety of Adaptive Mechanisms to Persist in Their Hosts. Int. J. Mol. Sci..

[132] Edwards R.A., Vega A.A., Norman H.M., Ohaeri M., Levi K., Dinsdale E.A., Cinek O., Aziz R.K., McNair K., Barr J.J. (2019). Global phylogeography and ancient evolution of the widespread human gut virus crAssphage. Nat. Microbiol..

[133] Yang J., Li Y., Wen Z., Liu W., Meng L., Huang H. (2021). Oscillospira—A candidate for the next-generation probiotics. Gut Microbes.

[134] Wu Y.T., Shen S.J., Liao K.F., Huang C.Y. (2022). Dietary plant and animal protein sources oppositely modulate fecal *Bilophila* and *Lachnoclostridium* in vegetarians and omnivores. Microbiol. Spectr..

[135] Yang J., Yu J. (2018). The association of diet, gut microbiota and colorectal cancer: What we eat may imply what we get. Protein Cell.

[136] Saha B., AT R., Adhikary S., Banerjee A., Radhakrishnan A.K., Duttaroy A.K., Pathak S. (2024). Exploring the relationship between diet, lifestyle and gut microbiome in colorectal cancer development: A recent update. Nutr. Cancer.

[137] Mogotsi M.T., Ogunbayo A.E., Bester P.A., O’Neill H.G., Nyaga M.M. (2024). Longitudinal analysis of the enteric virome in paediatric subjects from the Free State Province, South Africa, reveals early gut colonisation and temporal dynamics. Virus Res..

[138] Nwokorogu V.C., Pillai S., San J.E., Pillay C., Nyaga M.M., Sabiu S. (2024). A metagenomic investigation of the faecal RNA virome structure of asymptomatic chickens obtained from a commercial farm in Durban, KwaZulu-Natal province, South Africa. BMC Genom..

[139] Chauhan R.P., San J.E., Gordon M.L. (2022). Metagenomic analysis of RNA fraction reveals the diversity of swine oral virome on South African backyard swine farms in the uMgungundlovu district of KwaZulu-Natal province. Pathogens.

[140] Dridi B., Henry M., El Khechine A., Raoult D., Drancourt M. (2009). High prevalence of *Methanobrevibacter smithii* and *Methanosphaera stadtmanae* detected in the human gut using an improved DNA detection protocol. PLoS ONE.

[141] Blais Lecours P., Marsolais D., Cormier Y., Berberi M., Haché C., Bourdages R., Duchaine C. (2014). Increased prevalence of *Methanosphaera stadtmanae* in inflammatory bowel diseases. PLoS ONE.

[142] Hoegenauer C., Hammer H.F., Mahnert A., Moissl-Eichinger C. (2022). Methanogenic archaea in the human gastrointestinal tract. Nat. Rev. Gastroenterol. Hepatol..

[143] Gaci N., Borrel G., Tottey W., O’Toole P.W., Brugère J.F. (2014). Archaea and the human gut: New beginning of an old story. World J. Gastroenterol. WJG.

[144] Ramezani A., Nolin T.D., Barrows I.R., Serrano M.G., Buck G.A., Regunathan-Shenk R., West R.E., Latham P.S., Amdur R., Raj D.S. (2018). Gut colonization with methanogenic archaea lowers plasma trimethylamine N-oxide concentrations in apolipoprotein e−/− mice. Sci. Rep..

[145] Low A., Lee J.K.Y., Gounot J.S., Ravikrishnan A., Ding Y., Saw W.Y., Tan L.W.L., Moong D.K.N., Teo Y.Y., Nagarajan N. (2022). Mutual exclusion of *Methanobrevibacter* species in the human gut microbiota facilitates directed cultivation of a *Candidatus Methanobrevibacter intestini* representative. Microbiol. Spectr..

[146] Cai M., Tang X. (2022). Human archaea and associated metabolites in health and disease. Biochemistry.

[147] Hoffmann C., Dollive S., Grunberg S., Chen J., Li H., Wu G.D., Lewis J.D., Bushman F.D. (2013). Archaea and fungi of the human gut microbiome: Correlations with diet and bacterial residents. PLoS ONE.

[148] Rashed R., Valcheva R., Dieleman L.A. (2022). Manipulation of gut microbiota as a key target for Crohn’s disease. Front. Med..

[149] Ghavami S.B., Rostami E., Sephay A.A., Shahrokh S., Balaii H., Aghdaei H.A., Zali M.R. (2018). Alterations of the human gut *Methanobrevibacter smithii* as a biomarker for inflammatory bowel diseases. Microb. Pathog..

[150] White J.F. (2017). Syntrophic imbalance and the etiology of bacterial endoparasitism diseases. Med. Hypotheses.

[151] Herrnreiter C.J., Murray M.G., Luck M., Ganesa C., Kuprys P.V., Li X., Choudhry M.A. (2025). Bacterial dysbiosis and decrease in SCFA correlate with intestinal inflammation following alcohol intoxication and burn injury. eGastroenterology.

[152] Kumpitsch C., Fischmeister F.P.h.S., Mahnert A., Lackner S., Wilding M., Sturm C., Springer A., Madl T., Holasek S., Högenauer C. (2021). Reduced B12 uptake and increased gastrointestinal formate are associated with archaeome-mediated breath methane emission in humans. Microbiome.

[153] Mohammadzadeh R., Mahnert A., Shinde T., Kumpitsch C., Weinberger V., Schmidt H., Moissl-Eichinger C. (2025). Age-related dynamics of predominant methanogenic archaea in the human gut microbiome. BMC Microbiol..

[154] Orgler E., Baumgartner M., Duller S., Kumptisch C., Hausmann B., Moser D., Khare V., Lang M., Köcher T., Frick A. (2024). Archaea influence composition of endoscopically visible ileocolonic biofilms. Gut Microbes.

[155] Ianiro G., Iorio A., Porcari S., Masucci L., Sanguinetti M., Perno C.F., Gasbarrini A., Putignani L., Cammarota G. (2022). How the gut parasitome affects human health. Ther. Adv. Gastroenterol..

[156] Wang Y., Waters A.K., Basalirwa G., Ssetaala A., Mpendo J., Namuniina A., Keneema E., Kiiza D., Kyosiimire-Lugemwa J., Mayanja Y. (2025). Impact of *Schistosoma mansoni* Infection on the Gut Microbiome and Hepatitis B Vaccine Immune Response in Fishing Communities of Lake Victoria, Uganda. Vaccines.

[157] Nieves-Ramírez M.E., Partida-Rodríguez O., Laforest-Lapointe I., Reynolds L.A., Brown E.M., Valdez-Salazar A., Morán-Silva P., Rojas-Velázquez L., Morien E., Parfrey L.W. (2018). Asymptomatic intestinal colonization with protist Blastocystis is strongly associated with distinct microbiome ecological patterns. Msystems.

[158] Audebert C., Even G., Cian A., Loywick A., Merlin S., Viscogliosi E., Chabé M. (2016). Colonization with the enteric protozoa Blastocystis is associated with increased diversity of human gut bacterial microbiota. Sci. Rep..

[159] O’Brien Andersen L., Karim A.B., Roager H.M., Vigsnæs L.K., Krogfelt K.A., Licht T.R., Stensvold C.R. (2016). Associations between common intestinal parasites and bacteria in humans as revealed by qPCR. Eur. J. Clin. Microbiol. Infect. Dis..

[160] Beghini F., Pasolli E., Truong T.D., Putignani L., Cacciò S.M., Segata N. (2017). Large-scale comparative metagenomics of Blastocystis, a common member of the human gut microbiome. ISME J..

[161] Adeleke O.A., Yogeswaran P., Wright G. (2015). Intestinal helminth infections amongst HIV-infected adults in Mthatha General Hospital, South Africa. Afr. J. Prim. Health Care Fam. Med..

[162] Mkhize-Kwitshana Z.L., Tadokera R., Mabaso M.H. (2017). Helminthiasis: A systematic review of the immune interactions present in individuals coinfected with HIV and/or tuberculosis. Human Helminthiasis.

[163] Mpaka-Mbatha M.N., Naidoo P., Islam M.M., Singh R., Mkhize-Kwitshana Z.L. (2023). Demographic profile of HIV and helminth-coinfected adults in KwaZulu-Natal, South Africa. S. Afr. J. Infect. Dis..

[164] Chabé M., Certad G., Caccio S.M. (2021). Enteric unicellular eukaryotic parasites and gut microbiota: Mechanisms and mcology. Front. Microbiol..

[165] Laforest-Lapointe I., Arrieta M.C. (2018). Microbial eukaryotes: A missing link in gut microbiome studies. mSystems.

[166] Lukeš J., Stensvold C.R., Jirků-Pomajbíková K., Wegener Parfrey L. (2015). Are human intestinal eukaryotes beneficial or commensals?. PLoS Pathog..

[167] Zhang Y., Tan P., Zhao Y., Ma X. (2022). Enterotoxigenic Escherichia coli: Intestinal pathogenesis mechanisms and colonization resistance by gut microbiota. Gut Microbes.

[168] Sausset R., Petit M.A., Gaboriau-Routhiau V., De Paepe M. (2020). New insights into intestinal phages. Mucosal Immunol..

[169] Mukhopadhya I., Segal J.P., Carding S.R., Hart A.L., Hold G.L. (2019). The gut virome: The ‘missing link’ between gut bacteria and host immunity?. Ther. Adv. Gastroenterol..

[170] Zhao Z., Zhong L., Wu J., Zeng G., Liu S., Deng Y., Zhang Y., Tang X., Zhang M. (2025). Modulation of gut mycobiome and serum metabolome by a MUFA-rich diet in Sprague Dawley rats fed a high-fructose, high-fat diet. Foods.

[171] Mercer E.M., Ramay H.R., Moossavi S., Laforest-Lapointe I., Reyna M.E., Becker A.B., Simons E., Mandhane P.J., Turvey S.E., Moraes T.J. (2024). Divergent maturational patterns of the infant bacterial and fungal gut microbiome in the first year of life are associated with inter-kingdom community dynamics and infant nutrition. Microbiome.

[172] Bassetti M., Bandera A., Gori A. (2020). Therapeutic potential of the gut microbiota in the management of sepsis. Crit. Care.

[173] Gubatan J., Holman D.R., Puntasecca C.J., Polevoi D., Rubin S.J., Rogalla S. (2021). Antimicrobial peptides and the gut microbiome in inflammatory bowel disease. World J. Gastroenterol..

[174] Calvo-Barreiro L., Zhang L., Abdel-Rahman S.A., Naik S.P., Gabr M. (2023). Gut microbial-derived metabolites as immune modulators of T helper 17 and regulatory T cells. Int. J. Mol. Sci..

[175] Campbell C., Kandalgaonkar M.R., Golonka R.M., Yeoh B.S., Vijay-Kumar M., Saha P. (2023). Crosstalk between gut microbiota and host immunity: Impact on inflammation and immunotherapy. Biomedicines.

[176] He F., Zheng Y., Elsabagh M., Fan K., Zha X., Zhang B., Wang M., Zhang H. (2025). Gut microbiota modulate intestinal inflammation by endoplasmic reticulum stress-autophagy-cell death signaling axis. J. Anim. Sci. Biotechnol..

[177] Abou Diwan M., Lahimer M., Bach V., Gosselet F., Khorsi-Cauet H., Candela P. (2023). Impact of pesticide residues on the gut-microbiota–blood–brain barrier Axis: A narrative review. Int. J. Mol. Sci..

[178] Alagiakrishnan K., Morgadinho J., Halverson T. (2024). Approach to the diagnosis and management of dysbiosis. Front. Nutr..

[179] Kopczyńska J., Kowalczyk M. (2024). The potential of short-chain fatty acid epigenetic regulation in chronic low-grade inflammation and obesity. Front. Immunol..

[180] Bolte L.A., Vila A.V., Imhann F., Collij V., Gacesa R., Peters V., Wijmenga C., Kurilshikov A., Campmans-Kuijpers M.J., Fu J. (2021). Long-term dietary patterns are associated with pro-inflammatory and anti-inflammatory features of the gut microbiome. Gut.

[181] Hetta H.F., Ramadan Y.N., Alharbi A.A., Alsharef S., Alkindy T.T., Alkhamali A., Albalawi A.S., El Amin H. (2024). Gut microbiome as a target of intervention in inflammatory bowel disease pathogenesis and therapy. Immuno.

[182] Recharla N., Geesala R., Shi X.Z. (2023). Gut microbial metabolite butyrate and its therapeutic role in inflammatory bowel disease: A literature review. Nutrients.

[183] Mohan V.K., George M. (2021). A review of the contribution of gut-dependent microbiota derived marker, trimethylamine N-oxide (TMAO), in coronary artery disease. Curr. Res. Nutr. Food Sci. J..

[184] Jawhara S. (2022). How gut bacterial dysbiosis can promote Candida albicans overgrowth during colonic inflammation. Microorganisms.

[185] Soliman N., Kruithoff C., San Valentin E.M., Gamal A., McCormick T.S., Ghannoum M. (2025). Small Intestinal Bacterial and Fungal Overgrowth: Health Implications and Management Perspectives. Nutrients.

[186] Houtman T.A., Eckermann H.A., Smidt H., de Weerth C. (2022). Gut microbiota and BMI throughout childhood: The role of firmicutes, bacteroidetes, and short-chain fatty acid producers. Sci. Rep..

[187] Karačić A., Renko I., Krznarić Ž., Klobučar S., Liberati Pršo A.M. (2024). The Association between the Firmicutes/Bacteroidetes Ratio and Body Mass among European Population with the Highest Proportion of Adults with Obesity: An Observational Follow-Up Study from Croatia. Biomedicines.

[188] Mthombeni T.C., Burger J.R., Lubbe M.S., Julyan M. (2022). Antibiotic consumption in the public sector of the Limpopo province, South Africa, 2014–2018. S. Afr. J. Infect. Dis..

[189] Ferreira M., Cronjé H.T., Van Zyl T., Bondonno N., Pieters M. (2022). The association between an energy-adjusted dietary inflammatory index and inflammation in rural and urban Black South Africans. Public Health Nutr..

[190] Nkambule S.J., Moodley I., Kuupiel D., Mashamba-Thompson T.P. (2021). Association between food insecurity and key metabolic risk factors for diet-sensitive non-communicable diseases in sub-Saharan Africa: A systematic review and meta-analysis. Sci. Rep..

[191] Hou K., Wu Z.X., Chen X.Y., Wang J.Q., Zhang D., Xiao C., Zhu D., Koya J.B., Wei L., Li J. (2022). Microbiota in health and diseases. Signal Transduct. Target. Ther..

[192] Toor D., Wasson M.K., Kumar P., Karthikeyan G., Kaushik N.K., Goel C., Singh S., Kumar A., Prakash H. (2019). Dysbiosis disrupts gut immune homeostasis and promotes gastric diseases. Int. J. Mol. Sci..

[193] Khaledi M., Poureslamfar B., Alsaab H.O., Tafaghodi S., Hjazi A., Singh R., Alawadi A.H., Alsaalamy A., Qasim Q.A., Sameni F. (2024). The role of gut microbiota in human metabolism and inflammatory diseases: A focus on elderly individuals. Ann. Microbiol..

[194] Kashyap S., Das A. (2023). Exploring the complex and multifaceted interplay of the gut microbiome and cancer prevention and therapy. Acta Microbiol. Immunol. Hung..

[195] Van Hul M., Cani P.D., Petitfils C., De Vos W.M., Tilg H., El-Omar E.M. (2024). What defines a healthy gut microbiome?. Gut.

[196] Farup P.G., Aasbrenn M., Valeur J. (2018). Separating “good” from “bad” faecal dysbiosis-evidence from two cross-sectional studies. BMC Obes..

[197] Singh R., Zogg H., Wei L., Bartlett A., Ghoshal U.C., Rajender S., Ro S. (2021). Gut microbial dysbiosis in the pathogenesis of gastrointestinal dysmotility and metabolic disorders. J. Neurogastroenterol. Motil..

[198] Pollak M. (2017). The effects of metformin on gut microbiota and the immune system as research frontiers. Diabetologia.

[199] Ma W., Chen J., Meng Y., Yang J., Cui Q., Zhou Y. (2018). Metformin alters gut microbiota of healthy mice: Implication for its potential role in gut microbiota homeostasis. Front. Microbiol..

[200] Petakh P., Kamyshna I., Oksenych V., Kainov D., Kamyshnyi A. (2023). Metformin therapy changes gut microbiota alpha-diversity in COVID-19 patients with type 2 diabetes: The role of SARS-CoV-2 variants and antibiotic treatment. Pharmaceuticals.

[201] Rojo D., Méndez-García C., Raczkowska B.A., Bargiela R., Moya A., Ferrer M., Barbas C. (2017). Exploring the human microbiome from multiple perspectives: Factors altering its composition and function. FEMS Microbiol. Rev..

[202] Lee J., Menon N., Lim C.T. (2024). Dissecting gut-microbial community interactions using a gut microbiome-on-a-chip. Adv. Sci..

[203] Tuddenham S., Sears C.L. (2015). The intestinal microbiome and health. Curr. Opin. Infect. Dis..

[204] Pereira L., Mutesa L., Tindana P., Ramsay M. (2021). African genetic diversity and adaptation inform a precision medicine agenda. Nat. Rev. Genet..

[205] Dostal A., Baumgartner J., Riesen N., Chassard C., Smuts C.M., Zimmermann M.B., Lacroix C. (2014). Effects of iron supplementation on dominant bacterial groups in the gut, faecal SCFA and gut inflammation: A randomised, placebo-controlled intervention trial in South African children. Br. J. Nutr..

[206] Mahdavinia M., Rasmussen H.E., Engen P., Van den Berg J.P., Davis E., Engen K., Green S.J., Naqib A., Botha M., Gray C. (2017). Atopic dermatitis and food sensitization in South African toddlers: Role of fiber and gut microbiota. Ann. Allergy Asthma Immunol..

[207] Claassen-Weitz S., Gardner-Lubbe S., Nicol P., Botha G., Mounaud S., Shankar J., Nierman W.C., Mulder N., Budree S., Zar H.J. (2018). HIV-exposure, early life feeding practices and delivery mode impacts on faecal bacterial profiles in a South African birth cohort. Sci. Rep..

[208] Van Niekerk M., Dunbar R., Benycoub J., Grathwohl D., Labadarios D. (2019). Microbiota Richness and Diversity in a Cohort of Underweight HIV-Positive Children Aged 24–72 Months in Cape Town, South Africa. HIV Med..

[209] Budree S., Osman M., Nduru P., Kaba M., Zellmer C., Claasens S., Zar H. (2018). Evaluating the gut microbiome in children with stunting: Findings from a South African birth cohort. Am. J. Trop. Med. Hyg..

[210] Krishnamoorthy S., Coetzee V., Kruger J., Potgieter H., Buys E.M. (2020). Dysbiosis signatures of fecal microbiota in South African infants with respiratory, gastrointestinal, and other diseases. J. Pediatr..

[211] Goosen C., Proost S., Baumgartner J., Mallick K., Tito R.Y., Barnabas S.L., Cotton M.F., Zimmermann M.B., Raes J., Blaauw R. (2023). Associations of HIV and iron status with gut microbiota composition, gut inflammation and gut integrity in South African school-age children: A two-way factorial case-control study. J. Hum. Nutr. Diet..

[212] Van Zyl K., Whitelaw A.C., Hesseling A.C., Seddon J.A., Demers A.M., Newton-Foot M. (2021). Association between clinical and environmental factors and the gut microbiota profiles in young South African children. Sci. Rep..

[213] Wallenborn J.T., Gunier R.B., Pappas D.J., Chevrier J., Eskenazi B. (2021). Breastmilk, Stool, and Meconium: Bacterial Communities in South Africa. Microb. Ecol..

[214] Fei N., Bernabé B.P., Lie L., Baghdan D., Bedu-Addo K., Plange-Rhule J., Forrester T.E., Lambert E.V., Bovet P., Gottel N. (2019). The human microbiota is associated with cardiometabolic risk across the epidemiologic transition. PLoS ONE.

[215] Chen T., Long W., Zhang C., Liu S., Zhao L., Hamaker B.R. (2017). Fiber-utilizing capacity varies in Prevotella-versus Bacteroides-dominated gut microbiota. Sci. Rep..

[216] Bartsch M., Vital M., Woltemate S., Bouwman F.G., Berkemeyer S.B., Hahn A., Müller M. (2025). Microbiota-dependent fiber responses: A proof-of-concept study on short-chain fatty acid production in Prevotella-and Bacteroides-dominated healthy individuals. J. Nutr..

[217] Beam A., Clinger E., Hao L. (2021). Effect of diet and dietary components on the composition of the gut microbiota. Nutrients.

[218] Greco L., Rubbino F., Ferrari C., Michela C., Grizzi F., Bonelli F., Malesci A., Mazzone M., Ricciardiello L., Laghi L. (2025). Association of Fusobacterium nucleatum with colorectal cancer molecular subtypes and its outcome. A systematic review. Gut Microbiome.

[219] Daunizeau C., Franck M., Boutin A., Ruel M., Poliakova N., Ayotte P., Bélanger R. (2025). The gut microbiota of Indigenous populations in the context of dietary westernization: A systematic review and meta-analysis. Front. Nutr..

[220] Hetta H.F., Sirag N., Elfadil H., Salama A., Aljadrawi S.F., Alfaifi A.J., Alwabisi A.N., AbuAlhasan B.M., Alanazi L.S., Aljohani Y.A. (2025). Artificial sweeteners: A double-edged sword for gut microbiome. Diseases.

[221] Ross F.C., Patangia D., Grimaud G., Lavelle A., Dempsey E.M., Ross R.P., Stanton C. (2024). The interplay between diet and the gut microbiome: Implications for health and disease. Nat. Rev. Microbiol..

[222] Reynolds A., Mann J., Cummings J., Winter N., Mete E., Te Morenga L. (2019). Carbohydrate quality and human health: A series of systematic reviews and meta-analyses. Lancet.

[223] Jorgensen J.A., Choo-Kang C., Wang L., Issa L., Gilbert J.A., Ecklu-Mensah G., Luke A., Bedu-Addo K., Forrester T., Bovet P. (2025). Toxic metals impact gut microbiota and metabolic risk in five African-origin populations. Gut Microbes Rep..

